# Integrating Veterinary Public Health Data into EPCIS-Based Digital Traceability for Dairy Supply Chains

**DOI:** 10.3390/foods15091566

**Published:** 2026-05-01

**Authors:** Stavroula Chatzinikolaou, Giannis Vassiliou, Mary Gianniou, Michalis Vassalos, Nikolaos Papadakis

**Affiliations:** 1 Department of Electrical and Computer Engineering, Hellenic Mediterranean University, 71410 Heraklion, Greece; s.chatzinikolaou@gmail.com (S.C.); vassil@hmu.gr (G.V.); vassalos_mich@yahoo.gr (M.V.); 2 Directorate of Veterinary Services of the Regional Unit of Lesvos, Department of Animal Health, 81100 Mytilini, Greece; 3 Ministry of Rural Development and Food, General Directorate of Veterinary Services, Directorate of Animal Health, 10176 Athens, Greece; marygianniou@gmail.com

**Keywords:** digital traceability, EPCIS, dairy supply chains, veterinary public health, food safety, agri-food informatics, supply chain transparency, regulatory compliance, recalls, food waste, outbreak containment, consumer protection

## Abstract

Dairy foods—particularly cheeses produced from raw or minimally processed milk—remain vulnerable to hazards such as Listeria monocytogenes, where delayed laboratory confirmation can expand recalls, increase food waste, and delay outbreak containment. This study proposes a veterinary-aware digital traceability framework that embeds herd health data, milk-quality testing, and inspection outcomes directly into batch-level EPCIS event records. By representing veterinary public health controls as structured, machine-actionable traceability elements, the framework enables automatic logging of mandatory control points, systematic compliance verification, and rule-based risk state transitions within standard EPCIS infrastructures. Using regulation-consistent dairy simulations modeling delayed Listeria detection during maturation, we evaluate the operational impact of event-level causal traceability within the proposed architecture. Compared with conventional time-window recall strategies, provenance-based trace-forward queries reduced recall scope under the evaluated synthetic scenarios. Integrating structured veterinary controls into EPCIS-based traceability systems supports automated regulatory evidence generation and more targeted recall decisions, contributing to improved auditability and reduced food waste in dairy supply chains.

## 1. Introduction

The globalization of agri-food supply chains, increased regulatory pressure, and heightened public concern over foodborne illnesses have made digital traceability a central pillar of modern food safety systems [1]. In particular, dairy products—such as cheese—face heightened scrutiny due to their biological sensitivity and potential as vectors for zoonotic pathogens. Within this landscape, digital traceability tools are increasingly employed to record processing stages such as milk reception, pasteurization, curd formation, maturation, and packaging. These tools are typically implemented using standardized event-capture models such as the EPCIS (Electronic Product Code Information Services) framework, which has gained traction for its interoperability and machine-readability across actors and systems.

Dairy production presents distinctive food-safety challenges that place specific demands on traceability system design. Products derived from raw or minimally processed milk may be exposed to a range of biological and chemical hazards, including zoonotic pathogens (e.g., *Listeria monocytogenes*, *Salmonella* spp., *Brucella* spp.), environmental contaminants, and veterinary drug residues [2]. Several of these hazards exhibit characteristics that complicate containment, including environmental persistence, growth under refrigeration, delayed laboratory confirmation, and transmission pathways spanning both farm-level and post-processing stages. In multi-stage transformation chains (milk → curd → cheese), contamination signals may emerge after distribution events have already occurred, increasing reliance on precautionary, time-window–based recalls [3,4]. Such recalls often extend beyond causally affected batches, contributing to unnecessary product withdrawal, food waste, and economic loss [3,5]. These structural features of dairy supply chains—coupling herd health, hygiene controls, and maturation-dependent risk dynamics—underscore the need for traceability systems capable of integrating upstream veterinary indicators with downstream product-level events in a unified, machine-actionable framework.

However, current implementations in the dairy sector overwhelmingly focus on product and logistics data—tracking movements, transformations, and batch aggregations—while omitting structured veterinary public health information. This omission is critical, as veterinary oversight plays a fundamental role in detecting zoonotic risks, ensuring herd-level biosecurity, and verifying hygiene compliance at both farm and processing stages. Pathogens such as *Listeria monocytogenes*, *Brucella* spp., and *Mycobacterium bovis* (bovine tuberculosis) remain persistent threats in cheese production, particularly in raw milk products. Recent surveillance studies continue to report the presence of *L. monocytogenes* in dairy products within European markets, underscoring the ongoing relevance of robust traceability and risk-containment infrastructures [6]. Yet, the traceability systems designed to detect and contain these threats often operate in parallel to, or disconnected from, veterinary control systems.

The absence of integrated animal-health data weakens outbreak response. It also limits risk-based decision-making, proactive recalls, and transparent regulatory reporting. While there are separate digital infrastructures for animal identification, vaccination tracking, and laboratory diagnostics, these are rarely connected to supply-chain event records in a structured or automated way. This results in a fragmented digital ecosystem in which food safety, public health, and traceability operate in silos—undermining the goals of integrated, interoperable oversight.

This paper addresses this systemic gap by proposing a system that integrates veterinary data into EPCIS-based event traceability for cheese production. The framework enables end-to-end modeling of milk and cheese batches in conjunction with animal health indicators, hygiene inspections, and zoonotic risk parameters. By aligning this model with EU Regulations 852/2004 and 853/2004, we provide a schema-level pathway toward interoperable, regulation-aware traceability systems that embed both process and public health oversight. This work adopts a design-oriented and pre-deployment validation perspective, rather than reporting results from operational deployment.

### 1.1. Evaluation Scope and Methodological Framing

This paper adopts a design-oriented systems research perspective to address a structural gap in digital dairy traceability. Rather than evaluating a single industrial deployment, the work focuses on the specification, integration, and validation of a standards-aligned computational framework that embeds veterinary public health controls directly into EPCIS-based traceability systems. Given the limited accessibility of operational traceability repositories, regulated veterinary datasets, and the practical difficulty of reproducing food-safety incidents under controlled conditions, the proposed framework is evaluated using synthetic but regulation-consistent and EPCIS-aligned datasets. This approach enables controlled validation of functional correctness, risk propagation across transformation events, compliance verification, and operational feasibility, while remaining aligned with realistic dairy production constraints.

### 1.2. Separation of Contributions and Validation Scope

The proposed framework comprises three complementary contributions that are supported by different levels of evidence. First, the data-modeling contribution provides a standards-aligned representation that integrates veterinary public health information into EPCIS event structures. This component is conceptual and design-oriented, focusing on schema mappings and interoperability.

Second, the compliance-automation capability introduces rule-based mechanisms for verifying mandatory control points and regulatory constraints. This component is illustrated through formal definitions and example constraints, but is not evaluated through large-scale deployment.

Third, the recall-optimization aspect is evaluated using synthetic, regulation-consistent scenarios, where provenance-based traceability and risk propagation are used to analyze recall scope under controlled conditions. The quantitative results reported in this paper should therefore be interpreted within this simulated context.

This separation clarifies that while the framework contributes to multiple aspects of digital traceability, the level of empirical validation differs across components.

### 1.3. Novel Contributions

Research on food traceability and veterinary informatics has progressed substantially over the past two decades; however, these domains have largely evolved in parallel. EPCIS-based approaches primarily focus on product transformations, logistics events, and interoperability across supply-chain actors, while veterinary information systems emphasize animal health monitoring, disease surveillance, and regulatory reporting. As a result, structured veterinary public health data are rarely represented as first-class elements within product-level traceability systems.

To address this gap, this paper proposes a veterinary-aware extension of EPCIS-based digital traceability for dairy supply chains. The contributions of this work are design-oriented and span data modeling, system architecture, and governance considerations. Specifically, the paper makes the following contributions:

- **Data model contribution:** A standards-aligned representation that embeds veterinary public health controls—such as inspection checkpoints, milk-quality indicators, zoonotic risk signals, and vaccination status—into EPCIS event structures using Common Business Vocabulary (CBV) extensions and schema mappings;
- **Compliance and traceability integration:** A structured approach for representing regulatory control points and compliance-relevant events within EPCIS, enabling systematic verification of hygiene and veterinary requirements as part of the traceability record;
- **Governance-by-design framework:** A privacy-aware data-governance approach that supports cross-organizational traceability through pseudonymisation, attribute minimisation, and role-based access control, consistent with GDPR-oriented principles;
- **System architecture:** A hybrid traceability architecture combining relational storage with selective semantic processing, designed to support both operational queries and compliance-oriented reasoning;
- **Exploratory evaluation of recall behavior:** A synthetic, regulation-consistent evaluation illustrating how provenance-based traceability and risk propagation mechanisms influence recall scope under controlled scenarios.

Taken together, these contributions provide a structured foundation for integrating veterinary public health information into digital traceability systems while highlighting the potential and limitations of such integration in pre-deployment settings.

By bridging regulatory, veterinary, and digital infrastructure domains, the framework lays the foundation for traceability systems that are not only process-transparent but also health-aware, risk-resilient, and legally compliant.

The remainder of the paper is structured as follows. Section 2 reviews existing work on EPCIS-based traceability, veterinary informatics, and data governance. Section 3 formally defines the problem and presents the proposed solution approach. Section 4 details the system and mapping of veterinary concepts to EPCIS structures. Section 5 provides an illustrative cheese supply-chain scenario demonstrating targeted risk detection and recall scoping using the proposed event-level linkage. Section 6 discusses cases when mechanisms by which structured veterinary controls can constrain recall scope. Section 7 presents a synthetic evaluation demonstrating risk propagation, compliance verification, and recall scope reduction. Finally, Section 8 discusses implementation challenges and future work, and Section 9 concludes.

## 2. Related Work

Research on food traceability has expanded over two decades, driven by disease outbreaks, regulatory pressure, and consumer transparency demands. While traceability provides safety assurance, process optimization, and economic benefits for perishable products like dairy [7], many SMEs view it primarily as a compliance burden rather than an efficiency enabler [7,8]. Dairy ICT approaches include ontology-driven systems modeling product transformations and quality attributes [9], plus RFID, IoT sensors, and integrated monitoring platforms [10,11]. However, these treat veterinary and animal-health information as exogenous inputs or aggregate indicators rather than first-class traceability entities.

GS1 standards, particularly EPCIS, provide interoperable event-capture layers recording “what, when, where, and why” about product movements, enabling end-to-end visibility and targeted recalls [12]. Methodologies for modeling traceability information using EPCIS demonstrate standardized representation of critical tracking events across heterogeneous actors [13]. Case studies in meat and dairy show EPCIS supports multi-tier aggregation, item-level tracking, and consumer services via RFID and barcodes [8,10]. Yet implementations focus on product and logistics data (identifiers, locations, transformations, shipping) and seldom incorporate detailed veterinary controls (herd health status, zoonosis risk, inspection outcomes), leaving animal-health surveillance only partially aligned with product-level traceability.

Parallel work on animal disease traceability and digital health data transformation remains loosely connected to GS1-based food traceability. Policy documents from the USDA and the EU stress that animal and animal-product traceability is critical for outbreak management and trade facilitation. Technical roadmaps advocate integrated, multi-purpose recording systems supporting breeding, disease surveillance, veterinary drug use, and food-safety monitoring under “One Health” frameworks, highlighting fragmentation in animal health databases and integration challenges across veterinary practices, laboratories, and authorities. These rarely specify how veterinary data could be encoded using event-based standards like EPCIS or linked to downstream food products and processing.

Blockchain and IoT approaches argue that distributed ledgers enhance immutability and transparency when combined with IoT sensing [11,14,15,16]. Dairy prototypes use sensors for cold-chain tracking, while smart contracts and tokenized identifiers represent batches and certificates [17,18]. Even these sophisticated systems limit veterinary control integration to high-level flags (“fit for consumption”) or external certificate references without structured representation of underlying health and inspection events. Our approach explicitly models veterinary controls as structured EPCIS attributes and event extensions, enabling machine-readable links between animal health information, process steps, and regulatory states.

Data governance and privacy for veterinary and farm data are still not well defined. The GDPR requires strict rules for handling personal and health-related data linked to identifiable owners or locations [19]. Veterinary organizations give general advice about data protection, but this usually applies to single clinics, not to larger systems where data is shared across many organizations. In the food sector, discussions about governance often focus on protecting business information rather than sensitive veterinary data [7,8]. This creates a gap between general GDPR rules and practical database designs that allow storing only necessary data, controlling who can access it, and using pseudonyms to protect identities.

Conceptual frameworks for digital food ecosystems propose ways to share data across multiple organizations [20], including federated access, role-based permissions, and privacy-friendly analytics [10,17]. They emphasize using standard identifiers, aligning meanings across datasets (semantic harmonization), and clearly defining who controls the data and who processes it. However, these frameworks are often high-level and do not describe specific event formats or database schemas that can be directly implemented in a given supply chain. Our work addresses this gap by providing a concrete, domain-specific implementation: we map veterinary control activities to EPCIS events and relational database schemas, and we operationalize governance practices (e.g., collecting only necessary attributes and using pseudonyms) through master data tables and extension fields.

In summary, existing research progresses along three parallel tracks: EPCIS-based food traceability, animal health digital infrastructures, and data governance discussions. Missing is a unified, implementable model embedding veterinary controls into EPCIS event structures compatible with food-safety regulation and GDPR-oriented governance. This paper fills this gap through concrete mappings instantiable in pilot deployments and quantitatively evaluable in future work.

### Positioning Against Existing EPCIS Deployments

A practical implementation of EPCIS in agri-food supply chains is presented by Kassahun et al. [21], who deployed a cloud-based EPCIS architecture to enable chain-wide transparency in meat supply chains. Their work (Table 1) demonstrates real-world event capture, inter-organizational data exchange, and traceability query functionality using GS1 standards. However, the focus remains on product movement, aggregation, and transformation events without structured encoding of veterinary public health controls or rule-based risk propagation mechanisms.

In contrast, the present system extends EPCIS 2.0 and Common Business Vocabulary (CBV) constructs to represent herd-health context, milk-quality indicators, zoonotic risk classifications, and inspection outcomes as machine-actionable event attributes. Beyond transparency and interoperability, the proposed architecture enables automated compliance verification and quantitative recall-scope optimization through provenance-aware trace-forward queries. The contribution, therefore, represents a standards-aligned extension of existing EPCIS infrastructures that embeds veterinary intelligence and risk reasoning directly into operational traceability systems rather than proposing an alternative platform.

## 3. Problem Statement and Proposed Solution

### 3.1. Problem Statement

Digital traceability systems in agri-food supply chains are increasingly used to support transparency, compliance, and food safety [1]. In dairy production, and particularly in cheese manufacturing, these systems typically capture product and logistics events (e.g., receiving, transformation, aggregation, shipping) using interoperable event models such as EPCIS. However, in most practical deployments, veterinary public health information remains stored in separate systems (e.g., farm veterinary records, inspection databases, laboratory information systems), and is not represented as structured, machine-actionable traceability data linked to product lots and transformations [22].

This separation creates a structural limitation for risk-informed operations in dairy supply chains. First, zoonotic and hygiene-related risk indicators (e.g., herd health status, milk quality results, inspection outcomes, and delayed laboratory detections) cannot be systematically propagated along derivation links from upstream milk lots to downstream cheese batches and distribution units. Second, compliance verification often relies on parallel documentation or manual cross-checking rather than on auditable, event-level evidence embedded in the traceability record [7,8,23]. Third, the lack of semantic alignment across heterogeneous veterinary and traceability data sources limits interoperability, automated reasoning, and regulator-ready reporting [12].

In summary, current EPCIS-based dairy traceability platforms provide strong support for “what/when/where/why” of product events, but do not natively encode veterinary public health controls as first-class entities within the event stream. As a result, outbreak response, recall scoping, and compliance checks tend to be slower, broader, and more manual than necessary when health signals emerge upstream or with a delay.

### 3.2. Design Goals and Requirements

To address this gap, an integrated framework should satisfy the following requirements:

1. **Event-level linkage between veterinary controls and product provenance:** Veternary checkpoints and health indicators should be linkable to lots/batches and to transformation/aggregation relationships so that risk context can travel along the derivation graph.
2. **Standards alignment and interoperability:** The approach should remain compatible with EPCIS and controlled vocabularies (e.g., CBV), enabling adoption without replacing existing traceability infrastructure.
3. **Automatable compliance evidence:** Mandatory controls (e.g., quality testing, inspections) should be representable as structured events and metadata so they can be queried and checked systematically.
4. **Operational feasibility at scale:** The solution must support near-real-time ingestion and typical traceability query workloads, without requiring full semantic reasoning for all operational queries.
5. **Governance and privacy by design:** Sensitive farm- and veterinary-related information must be supported under GDPR-oriented principles such as attribute minimisation, pseudonymisation, and layered access.

### 3.3. Proposed Solution Overview

We propose a system that embeds structured veterinary public health controls directly into EPCIS-based digital traceability for cheese production. The core idea is to treat veterinary and hygiene-relevant information as part of the traceability record by (i) encoding it through EPCIS extension mechanisms and master data, and (ii) enabling semantic interoperability and automated checks via an ontology and rule layer integrated with a high-performance relational backend.

(1) Veterinary-aware EPCIS event enrichment.

The framework extends standard EPCIS event streams (ObjectEvents, TransformationEvents, AggregationEvents) with veterinary and quality-control attributes using a combination of:

- **Instance/Lot Master Data (ILMD)** to capture lot-level measurements such as milk quality indicators (e.g., somatic cell count, residue screening results);
- **Master data extensions** (e.g., party attributes) to represent supplier/farm-level health and vaccination context;
- **Event-level extensions and metadata** to record inspection outcomes, laboratory test references, and zoonotic risk parameters in a structured form;
- **CBV-aligned business steps and dispositions** to represent states such as active, blocked, or quarantine, supporting interoperable interpretation across actors.

This design preserves EPCIS compatibility while enabling health-aware traceability at the same granularity as production and logistics events.

(2) Risk propagation and compliance verification on the provenance graph.

By explicitly representing transformation and aggregation relationships, the framework supports trace-back and trace-forward queries that connect upstream health signals to downstream product units. In particular, when an adverse finding (e.g., delayed detection of a pathogen during maturation-stage testing) is recorded for a lot or batch, the corresponding risk status can be propagated along derivation links to all downstream entities, enabling targeted holds and recall scoping. In parallel, compliance checks can be expressed as verifiable constraints over the event history (e.g., the presence of required inspection/testing events at defined control points and appropriate dispositions before commissioning/shipping).

(3) Hybrid SQL–RDF/OWL Architecture for Food-Safety–Oriented Interoperability

To support routine dairy operations while enabling structured food-safety verification, the framework adopts a hybrid integration approach. Day-to-day traceability functions—such as batch tracking, transformation logging, and shipment queries—are executed directly within the relational database to maintain operational efficiency. Semantic (RDF/OWL) representations are generated selectively for workflows requiring automated validation of hygiene controls, risk escalation, and regulatory investigation following contamination events. This design ensures that compliance checks and outbreak-related reasoning can be performed without slowing core production processes.

Because dairy operators use heterogeneous ERP and database systems, the framework maps local data structures to a shared, standards-aligned model (EPCIS and CBV). This harmonization enables consistent interpretation of veterinary controls and batch-level evidence across farms, processors, laboratories, and competent authorities. The primary objective is to strengthen coordinated compliance oversight and recall decision-making without requiring structural changes to existing production systems.

(4) Governance and privacy-preserving configuration.

The framework is designed to support GDPR-compatible deployment patterns such as:

- **Attribute minimization** (keeping only necessary identifiers and references in shared events);
- **Pseudonymisation** (protecting farm identity resolution through controlled mappings);
- **Layered access control** (restricting sensitive veterinary attributes to authorized roles).

These measures allow veterinary public health information to be integrated into traceability without requiring indiscriminate disclosure across supply-chain actors.

### 3.4. Health-Aware Traceability Model for Dairy Safety (HATM)

Dairy supply chains are characterized by (i) multi-stage transformations (milk → curd → cheese → packaged units), (ii) delayed availability of laboratory results (e.g., microbiological confirmation during maturation), and (iii) mandatory hygiene and veterinary control points. In such settings, a food-safety signal detected at one stage must be propagated consistently to all downstream products derived from the affected lots/batches, enabling timely holds, targeted recalls, and regulator-ready evidence. To formalize this behavior in a manner aligned with dairy food-safety operations, we define a Health-Aware Traceability Model (HATM) that captures provenance, risk annotation, and compliance constraints over event-level traceability records.

#### 3.4.1. Provenance Structure

Let G=(V,E) be a directed provenance graph, where each node v∈V represents a traceable entity in the dairy chain (e.g., raw milk lot, intermediate curd lot, cheese batch, case/pallet), and each directed edge (u,v)∈E represents a derivation relation meaning that entity *v* is produced using (or contains) entity *u*. Edges are instantiated from EPCIS transformation and aggregation events, thus supporting both trace-back (upstream) and trace-forward (downstream) reasoning.

#### 3.4.2. Risk Annotation for Dairy Hazards

Each entity is associated with a food-safety risk level derived from veterinary public health indicators and quality-control findings (e.g., herd-health status, antibiotic residue screening, hygiene inspection outcomes, or delayed detection of *Listeria monocytogenes*). We define a risk-annotation function as follows:

(1) ρ:V→R,

where R={low,medium,high,critical} is an ordered domain. The risk value ρ(v) is updated when new evidence becomes available (e.g., a lab result recorded days after production).

#### 3.4.3. Risk Propagation Across Dairy Transformations

When a lot/batch is elevated in risk, all products derived from it must inherit at least that risk level, because they remain causally linked through the transformation chain. Let u⇝v denote reachability in *G* (i.e., there exists a directed path from *u* to *v*). Risk propagates monotonically along provenance:

(2) ρ(v)←max{ρ(u)|u⇝v},

where max(·) is taken with respect to the ordering on *R*. Operationally, this captures the principle that a high-risk milk lot can induce quarantine/hold requirements for derived cheese batches and packaged units, even if the adverse signal is detected after distribution events have occurred.

#### 3.4.4. Compliance Constraints as Verifiable Predicates

In dairy production, regulatory and HACCP-oriented obligations require that specific controls occur at defined stages (e.g., reception checks, quality testing, hygiene inspection checkpoints, and release conditions prior to shipping). We model such obligations as a set of predicates:

(3) $$C=\{c_{1},\ldots ,c_{n}\},$$

where each $c_{i}\left(v\right)$ evaluates to true if the event history contains the evidence required for entity *v*.

**Example 1** (Mandatory milk quality test)**.**
*For a milk lot v, define:*

(4) $$c_{\mathrm{quality}}\left(v\right)\equiv \exists e\;\;s.t.\;e\;\mathrm{is}\;a\;\mathrm{recorded}\;\mathrm{quality}\text{-}\mathrm{testing}\;\mathrm{event}\;\mathrm{referencing}\;v\;\mathrm{prior}\;\mathrm{to}\;\mathrm{its}\;\mathrm{first}\;\mathrm{transformation}.$$

*If $c_{\mathrm{quality}}\left(v\right)$ is false, the lot is flagged as non-compliant.*

**Example 2** (Batch release constraint)**.**
*For a cheese batch v, define:*

(5) $$c_{\mathrm{release}}\left(v\right)\equiv \left(\rho (v)<\mathrm{high}\right)\;\wedge \;\mathrm{RequiredControlEventsPresent}\left(v\right),$$

*meaning that commissioning/shipping is permitted only if (i) risk is below a defined threshold and (ii) all required control-point events are present in the traceability record.*

#### 3.4.5. Model Definition and Operational Interpretation

The HATM is defined as the tuple:

(6) H=(G,ρ,C,P),

where *G* is the provenance graph, ρ is the risk annotation function, *C* is the set of compliance predicates, and *P* is the propagation operator induced by Equation (2). In practice, *G* and the evidence needed for *C* are instantiated from EPCIS events (Object, Transformation, Aggregation, and inspection/testing events or extensions), while *P* and selected compliance checks may be executed either via SQL-based provenance queries or via the RDF/OWL rule layer, depending on deployment needs.

This formalization is intentionally lightweight: it captures the essential food-safety behavior required for (i) propagating delayed hazard detections across milk-to-cheese derivation, (ii) verifying the presence of mandatory dairy hygiene/veterinary controls, and (iii) supporting targeted trace-forward recall decisions bounded to the affected provenance subgraph.

## 4. The Proposed System

### 4.1. Architecture Overview

**Figure 1:**
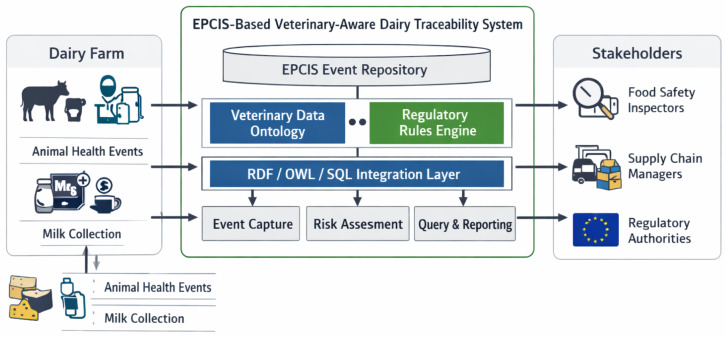
High-level architecture of the proposed EPCIS-based veterinary-aware dairy traceability system. Veterinary and quality-control data generated at farm and processing stages are encoded as EPCIS events and master data, enriched via an ontology and rule layer, and exposed through traceability queries and reporting to supply chain and regulatory stakeholders.

Figure 1 summarizes the architecture used to integrate veterinary public health controls into an EPCIS-based digital traceability system for dairy/cheese production (Figure 1).

#### 4.1.1. Data Sources (Farm and Production)

On the left, data originate from dairy farms and upstream veterinary systems (e.g., herd health status, vaccination records, inspection outcomes), as well as milk collection/reception activities. These data are represented in a structured form and linked to material identifiers (e.g., milk lots) so that animal-health context can be associated with downstream product lots/batches.

#### 4.1.2. EPCIS Event Repository (Core Traceability Layer)

At the center, an EPCIS event repository stores standard traceability events (e.g., ObjectEvents, TransformationEvents, AggregationEvents) describing the movement and transformation of milk into cheese and packaged units. Veterinary and quality attributes are attached using EPCIS extension mechanisms (e.g., ILMD and event extensions) and controlled vocabularies (CBV) to maintain interoperability.

#### 4.1.3. Semantic Integration and Rule Layer

To support semantic interoperability and automated checks, the architecture includes a veterinary data ontology and a regulatory/risk rules component. A hybrid integration layer bridges relational storage (SQL) and semantic representations (RDF/OWL), enabling traceability queries that exploit both transactional performance and graph-based reasoning over provenance and constraints.

#### 4.1.4. Operational Services

The lower components represent typical system services: (i) event capture/ingestion, (ii) risk assessment and risk propagation across transformation links (e.g., from a high-risk milk lot to derived cheese batches), and (iii) query and reporting functions for investigations, audits, and recall scoping.

#### 4.1.5. Stakeholder Access

On the right, different stakeholders consume the outputs of the system under appropriate governance controls: food safety inspectors, supply chain managers, and competent authorities. The architecture is intended to support targeted trace-back/trace-forward investigations, compliance verification at defined control points, and regulator-ready reporting using standardized event data.

The framework deliberately adopts EPCIS as the primary event model because it is supply-chain–native and widely deployed in food systems [12].

Alternative standards and modeling approaches were evaluated but found less suitable for batch-oriented, multi-actor dairy traceability [14,15,24].

In practice, the proposed architecture does not require full semantic reasoning on every traceability query. High-frequency operational queries (e.g., batch location, shipping status) remain SQL-based, while RDF/OWL reasoning is applied selectively for risk escalation, compliance verification, and investigative workflows. This separation limits computational overhead and aligns with typical dairy operational constraints.

### 4.2. Veterinary Data Integration Layer

This layer enables the structured capture of:

- Milk quality test results (e.g., somatic cell count, residues);
- Herd health status and animal welfare indicators;
- Zoonotic risk factors and vaccination records;
- Hygiene inspection outcomes at farm and processing levels.

These data elements are mapped to extended EPCIS event attributes using the Common Business Vocabulary, allowing standardized representation and exchange of veterinary information.

### 4.3. Event-Based Traceability Model

Key EPCIS event mappings in the framework include the following:

- **Object Events**: Identification of milk batch units linked to farm-level health records;
- **Transformation Events**: Conversion of milk into curd and cheese with inherited health metadata;
- **Aggregation Events**: Grouping of batches for storage, packaging, and distribution;
- **Verification Events (proposed extension)**: Recording of veterinary checkpoints at critical control points.

This event-based model enables traceability both upstream (animal → product) and downstream (product → exposure risk).

### 4.4. Regulatory Compliance Alignment

The framework operationalizes mandatory requirements from:

- **EU Regulation 852/2004**—General food hygiene obligations for food business operators;
- **EU Regulation 853/2004**—Specific hygiene rules for food of animal origin.

Instead of relying on parallel documentation systems, compliance information is embedded within digital event records, enhancing auditability, data integrity, and cross-actor communication.

### 4.5. Mapping Veterinary Concepts to EPCIS Structures

Veterinary public health concepts—including milk quality, herd health, and vaccination status, zoonosis indicators, and inspection outcomes—are encoded directly in the EPCIS event stream using three standard mechanisms: Instance/Lot Master Data (ILMD), event-level extensions, and master data attributes. This design treats veterinary signals as first-class, machine-actionable traceability elements while remaining fully compatible with EPCIS and the GS1 Common Business Vocabulary (CBV).

Milk quality measurements (e.g., somatic cell count, antibiotic residue screening, and compositional parameters) are attached to raw milk lots via ILMD on EPCIS ObjectEvents. For example, a milk reception event may include the following ILMD fragment linked to the corresponding lot identifier:


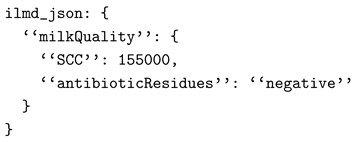


This information is stored at the lot level and remains associated with downstream products through explicit transformation relationships.

Herd health and vaccination context are maintained at the supplier (farm) level using EPCIS master data and linked to incoming lots via standard party-to-event associations. Zoonosis-related indicators and batch risk classifications are recorded at the lot level and surfaced operationally through CBV-compliant dispositions (e.g., active, blocked, quarantine), allowing health-related state changes to be interpreted consistently across actors.

Veterinary and hygiene checks at critical control points (e.g., farm audits, milk reception, and pre-maturation inspections) are represented as explicit EPCIS events enriched with structured inspection metadata, enabling them to be queried and audited alongside production and logistics events. Environmental measurements relevant to food safety (e.g., temperature and humidity) are linked through EPCIS sensor elements to integrate monitoring data without expanding core event structures.

Additional implementation-ready mappings and complete EPCIS payload examples are provided in Appendix A.

### 4.6. Mapping Regulatory Requirements to Business Processes

Mandatory hygiene controls from EU Regulations 852/2004 and 853/2004 are operationalized in the EPCIS model through standardized biz_step values, CBV dispositions and supporting metadata. This mapping turns regulatory obligations into verifiable constraints over the traceability event history, reducing reliance on external or document-based records.

Critical control points in cheese production—raw milk reception, processing, maturation, and batch release—are modeled using CBV-aligned biz_step values. Compliance outcomes are captured through dispositions (e.g., blocked, quarantined) and, where needed, complemented with structured justification metadata describing the non-compliance reason and any corrective action. References to certification and laboratory documentation are stored at the lot level so events can link to authoritative evidence without embedding sensitive documents in the event stream.

Together, these mappings embed compliance evidence within the same interoperable event framework used for operational traceability, supporting automated checks, auditability, and risk-informed decision-making. Detailed regulatory mappings and example EPCIS payloads are provided in Appendix A.

### 4.7. Summary Mapping Table

Table 2 summarizes the correspondence between key framework concepts and EPCIS constructs, with representative schema elements.

## 5. Illustrative Use Case: Targeted *Listeria* Risk Detection and Recall in a Cheese Supply Chain

### 5.1. Context and Objective

**Figure 2:**
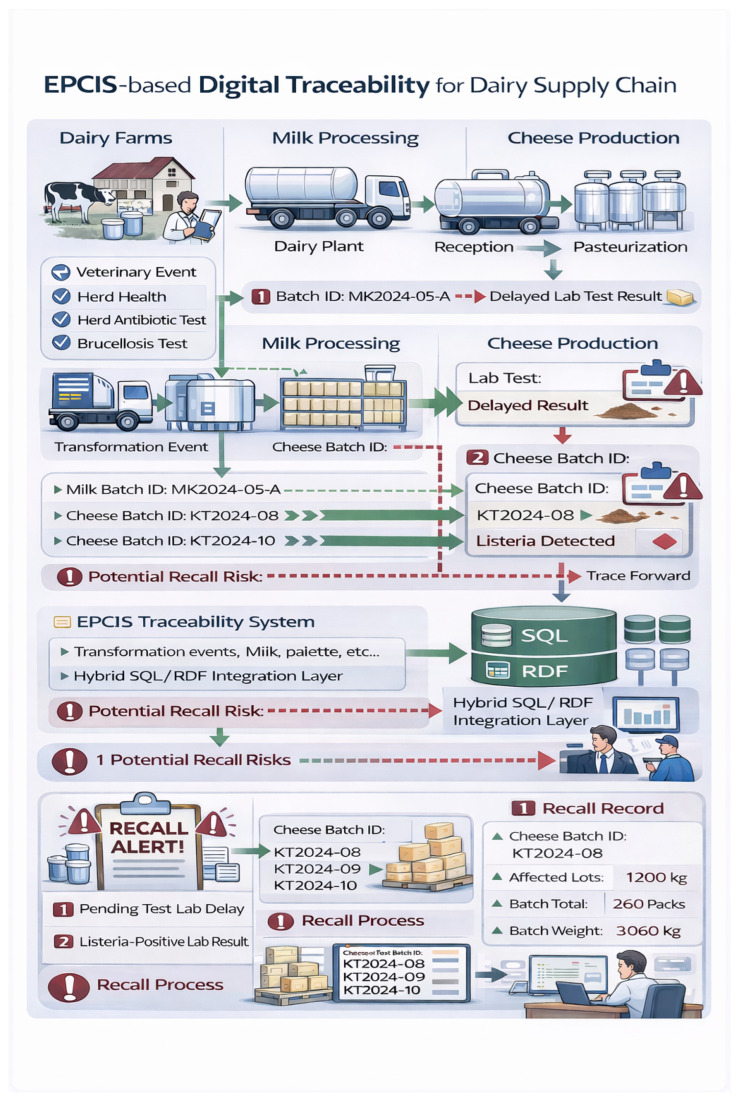
Illustration of Section 6 case study showing (1) veterinary context capture at milk reception, (2) transformation of milk lot into cheese batch, (3) delayed laboratory detection of Listeria monocytogenes, and (4) automated quarantine and recall activation. The figure highlights bidirectional traceability: trace-back from contaminated cheese batch to originating milk lot and farm, and trace-forward from the affected batch to derived distribution units. Explicit EPCIS transformation and aggregation events enable precise recall scoping, reducing unnecessary product withdrawal compared with time-window-based strategies.

This illustrative use case demonstrates (Figure 2) how the proposed EPCIS-based framework, extended with structured veterinary public health data, supports early risk detection, targeted recalls, and regulator-ready audit trails in a realistic cheese production scenario (Figure 2). The hazard considered is *Listeria monocytogenes*, a high-consequence pathogen for dairy products that is routinely monitored during processing and maturation [25,26,27,28]. The objective is to show (i) how the animal-health and quality-control context is bound to EPCIS event streams, and (ii) how event-based linkage enables rapid and precise scoping of affected products compared with conventional document-based tracing.

### 5.2. Actors and System Setup

The supply chain involves four actors:

- **Farm A**: A milk supplier subject to routine veterinary inspections and herd-health monitoring;
- **Dairy Plant B**: A processor receiving milk from multiple farms and producing batch-level cheese lots;
- **Laboratory C**: An accredited laboratory performing microbiological testing on milk and cheese samples;
- **Competent Authority D**: The regulatory body overseeing compliance, audits, and recall coordination.

Each actor operates an EPCIS-compatible repository or exposes EPCIS-compliant interfaces. Veterinary and laboratory systems remain operationally autonomous but publish structured summaries through EPCIS event extensions and master data. Access to sensitive farm- and veterinary-level attributes follows layered permissions consistent with GDPR-oriented data minimisation principles.

### 5.3. Step 1: Milk Reception with Veterinary Context Capture

On Day 0, Farm A delivers a raw milk lot (MilkLot-2024-05-A) to Dairy Plant B. At reception, Dairy Plant B records an EPCIS ObjectEvent with the following:

- bizStep = receiving;
- disposition = active;
- EPC identifiers corresponding to the received milk lot.

The event is enriched via Instance/Lot Master Data (ILMD) with routinely captured milk-quality indicators, such as somatic cell count (SCC), antibiotic residue screening, and compositional parameters. In parallel, Farm A’s herd-health and vaccination status are referenced through EPCIS party master data. At this stage, no non-compliance indicators are present.

### 5.4. Step 2: Processing and Risk Inheritance Through Transformation

On Day 1, MilkLot-2024-05-A is used to produce a cheese batch (CheeseBatch-2024-05-X). The processor records an EPCIS TransformationEvent linking:

- Input EPC: MilkLot-2024-05-A;
- Output EPC: CheeseBatch-2024-05-X.

In the proposed model, quality- and health-relevant metadata associated with the input lot remains contextually linked to the output batch through explicit transformation relationships. This enables consistent propagation of risk context along the milk-to-cheese derivation chain and supports downstream investigations that trace back to upstream veterinary checkpoints.

### 5.5. Step 3: Laboratory Detection and Automated Quarantine Disposition

On Day 14, during routine maturation-stage microbiological testing, Laboratory C detects *Listeria monocytogenes* in a sample associated with CheeseBatch-2024-05-X. The result is recorded as an EPCIS quality-testing event (e.g., an ObjectEvent or a domain-specific verification/inspection extension), including the following:

- bizStep = quality_testing (or an equivalent CBV-aligned step);
- A structured extension indicating detection of *Listeria monocytogenes*;
- A reference to the laboratory test identifier and sampling timestamp.

Upon detection, the batch disposition is updated to quarantine, and a risk assessment marker (e.g., risk = high) is attached to the batch metadata. In implementations using ontology rules or validation constraints, this update can automatically trigger downstream controls, such as blocking commissioning, preventing shipping events, and generating regulator notifications.

### 5.6. Operational Interpretation and Incident-Response Evidence Capture

Although the framework is presented as a standards-aligned digital traceability and reasoning model, the same EPCIS event stream can also encode and audit the first-response actions typically initiated following a *Listeria* detection, supporting both operational decision-making and regulator-ready evidence.

#### 5.6.1. Immediate Containment and Precautionary Holds

Following a positive finding linked to CheeseBatch-2024-05-X, Dairy Plant B applies a physical and system-enforced hold on all units of the affected batch across maturation, cold storage, and finished-goods areas. This action is recorded as a disposition update (e.g., quarantine or blocked) and/or as a dedicated incident or hold extension linked to the batch EPC. Where appropriate, precautionary holds may also be applied to exposure-linked lots (e.g., products sharing the same maturation room, equipment, or handling zones) pending confirmatory testing. These precautionary measures are operationally broader than the final recall boundary and are explicitly distinguishable within the event history.

#### 5.6.2. Rapid Traceability Capture for Scoping

At the time of detection, the system consolidates the essential batch context required for rapid investigation, including production date and time, product type, maturation room assignment, movement history, and—where available—intrinsic product parameters relevant to *Listeria* growth potential (e.g., pH, water activity, salt content). If distribution has occurred, recipient and shipment identifiers are linked through EPCIS aggregation and shipping events, enabling immediate trace-forward scoping.

#### 5.6.3. Verification Sampling and Corrective Actions

Verification sampling is performed to determine whether contamination comes from the product itself or from the surrounding environment. This may include testing products from different parts of the same batch, swabbing different areas of the facility (from food-contact surfaces to more distant areas), and testing liquids such as brine that are reused in the process.

If contamination is detected, corrective actions are taken. These may include additional cleaning and sanitizing of high-risk areas, followed by further testing to confirm that the cleaning was effective. All actions and test results are documented to provide clear evidence that the issue was properly addressed.

#### 5.6.4. Decision Logic: Containment Versus Recall

The model separates quick precautionary actions from full recalls.

If *Listeria monocytogenes* is confirmed in a product linked to the batch, a recall or product withdrawal is started. The affected products are identified using trace-forward records to find exactly where they were distributed.

If the bacteria are found only in the environment and product tests are negative, the situation is treated as a serious issue but not a recall. Corrective actions must be documented, monitoring is increased, and the batch can only be released if there is clear, time-stamped evidence showing the product is safe.

### 5.7. Step 4: Targeted Recall and Scope Reduction via EPCIS Linkage

Competent Authority D initiates trace-back and trace-forward analysis using EPCIS queries over event relationships:

- **Trace-back**: Identification of all upstream inputs and farm sources linked to CheeseBatch-2024-05-X;
- **Trace-forward**: Identification of all aggregated logistics units (e.g., pallets, cases) and shipment events derived from or containing the affected batch.

Because derivation and aggregation relationships are explicitly represented, the authority can isolate the affected scope to only those distribution units causally linked to the detected batch. Compared with time-window or facility-wide recalls, this event-based approach enables tighter recall boundaries, reducing unnecessary withdrawals, economic losses, and food waste while improving consumer protection.

### 5.8. Step 5: Regulatory Auditability and Compliance Verification

During follow-up inspection, auditors verify compliance obligations by reviewing the chronological EPCIS event history and associated inspection and testing metadata. This provides digital evidence that:

- Reception controls and milk-quality checks were recorded;
- Maturation-stage microbiological testing was performed as required;
- Quarantine dispositions and product holds were applied immediately upon detection;
- Commissioning and shipping constraints were enforced;
- Verification sampling and post-sanitation re-verification were initiated and documented.

The same event stream supports both operational decision-making (holds, targeted recalls) and regulatory reporting (inspection evidence, corrective actions), reducing reliance on manual cross-referencing between disconnected veterinary and processing record systems.

### 5.9. Use Case Summary

This scenario illustrates how embedding veterinary public health data within EPCIS event streams shifts traceability from passive record-keeping to an active food-safety decision-support mechanism. By unifying animal-health context, quality testing, incident-response evidence, and supply-chain transformations in interoperable events, the framework enables faster outbreak responsiveness, tighter recall scope definition, and more auditable compliance workflows.

## 6. Mechanisms by Which Structured Veterinary Controls Constrain Recall Scope

In real dairy operations, recall boundaries are frequently expanded not only because of missing causal traceability, but also due to incomplete documentation, uncertainty regarding upstream controls, or lack of verifiable hygiene evidence.

Structured integration of veterinary public health controls into the traceability record provides additional mechanisms that can constrain precautionary recall expansion under realistic regulatory conditions.

### 6.1. Batch-Level Risk Differentiation Through Documented Control Evidence

In conventional recall investigations, regulators may apply time-window or facility-wide heuristics when it is not possible to clearly differentiate between compliant and potentially non-compliant batches produced within the same operational period. When milk lots are not associated with structured, machine-verifiable veterinary control records (e.g., quality testing, herd-health status, inspection outcomes), precautionary logic may require inclusion of all temporally adjacent batches.

By encoding milk quality tests, inspection outcomes, and herd-health indicators directly within EPCIS events and lot-level metadata, each production batch can be evaluated against explicit, time-stamped control evidence. This enables authorities to distinguish between batches that have satisfied mandatory hygiene checkpoints and those with elevated or uncertain risk indicators. When contamination is epidemiologically linked to a specific milk lot or transformation chain, trace-forward analysis can be restricted to causally related batches, while contemporaneous but fully compliant batches remain excluded.

This discrimination capability reduces recall expansion driven by evidentiary uncertainty rather than causal linkage.

### 6.2. Prevention of Precautionary Expansion Due to Missing Documentation

A frequent cause of recall widening is incomplete or delayed documentation of mandatory controls. In the absence of verifiable evidence (e.g., milk reception testing records), authorities may extend recall boundaries as a precautionary measure.

When mandatory veterinary and hygiene checkpoints are represented as structured EPCIS events—such as quality-testing events at milk reception—compliance completeness can be automatically verified through traceability queries. Batches for which all required control events are present and time-aligned can be demonstrably excluded from the recall scope. Conversely, the absence of required checkpoints can be detected systematically and isolated to specific batches.

Thus, structured digital documentation reduces uncertainty-driven recall enlargement by transforming compliance verification from manual record inspection to machine-supported validation.

### 6.3. Constrained Environmental Exposure Boundaries

In dairy production, cross-contamination risks may be associated with defined processing lines, maturation rooms, brining systems, or sanitation cycles. When environmental monitoring and sanitation verification are not digitally linked to batch-level events, exposure may be conservatively assumed to affect entire facilities.

By associating inspection outcomes, environmental monitoring results, and sanitation verification events with defined processing locations and time intervals, the proposed framework allows more precise delineation of plausible exposure zones. This structured linkage enables regulators to differentiate between batches processed within documented risk compartments and those produced in verified clean environments.

Such spatially contextualized evidence supports recall decisions bounded to epidemiologically plausible exposure sets, rather than facility-wide assumptions.

### 6.4. Early Containment Through Automated Risk-State Transitions

Structured veterinary indicators recorded upstream—such as abnormal milk quality parameters, residue detection, or failed inspections—can trigger immediate disposition changes (e.g., *blocked* or *quarantine*) before products enter distribution channels.

In scenarios where risk signals are detected prior to commissioning or shipping events, automated state transitions can prevent potentially unsafe batches from entering commerce. While this mechanism does not reduce recall size after confirmed contamination, it reduces recall necessity by enabling earlier containment and preventing downstream distribution.

This shift from reactive recall to proactive containment represents a significant operational advantage in dairy safety management.

### 6.5. Illustrative Operational Scenario

To illustrate the combined effect of structured control documentation and causal traceability, consider a production day in which two cheese batches are produced on the same processing line within a 24 h window. Under a time-window recall heuristic, both batches may be withdrawn following a positive laboratory finding in one batch.

If the second batch is supported by complete, time-stamped veterinary inspection records, validated milk quality tests, and documented sanitation verification linked to its specific transformation chain, structured traceability evidence allows regulators to confine recall actions to the causally affected batch only. In such a scenario, recall scope reduction arises not solely from provenance linkage, but from demonstrable compliance completeness that eliminates precautionary uncertainty.

### 6.6. Boundary Conditions and Limitations

The constraining mechanisms described above depend on three operational conditions: (i) consistent capture of mandatory veterinary and hygiene events, (ii) timely ingestion of control data into the traceability repository, and (iii) reliable linkage between environmental compartments and batch identifiers. If these conditions are not satisfied, recall decisions may revert to broader provenance- or time-based heuristics.

Therefore, while event-level causal traceability provides the structural basis for targeted recall, structured veterinary control integration enhances this capability by improving evidentiary completeness, risk discrimination, and regulatory confidence.

### 6.7. Summary

Structured veterinary public health integration does not replace causal traceability as the primary mechanism of recall optimization. Rather, it strengthens recall boundary definition by reducing uncertainty, improving compliance transparency, and supporting earlier containment decisions. In combination, these mechanisms enable recall actions that are both epidemiologically justified and operationally proportionate, contributing to improved consumer protection and reduced food waste in dairy supply chains.

## 7. Evaluation

### 7.1. Practical Relevance and Scenario Grounding

Although the evaluation is based on synthetic datasets, the scenarios are constructed to reflect realistic dairy supply-chain conditions, including delayed laboratory confirmation, multi-stage transformations, and regulatory control points. These characteristics are consistent with documented dairy safety challenges, particularly in cheese production chains where contamination signals may emerge after distribution.

To further assess practical relevance, the modeled workflows and control points were aligned with standard veterinary inspection procedures and EU regulatory requirements. While a full industrial deployment is outside the scope of this work, the framework is designed to be directly compatible with existing EPCIS-based infrastructures, enabling future validation in operational environments.

### 7.2. Rationale for Synthetic, Regulation-Consistent Evaluation

Evaluating veterinary-integrated digital traceability in real dairy supply chains is constrained by legal, governance, and operational factors. Veterinary public health datasets are often linked—directly or indirectly—to identifiable persons, holdings, or locations. When such linkage enables identification, the data fall under GDPR and require strict purpose limitation, minimization, and legal authorization for secondary use. These constraints substantially restrict cross-organizational sharing of integrated veterinary and traceability records for research validation.

In addition to data protection concerns, traceability repositories contain commercially sensitive information [20], including supplier relationships, production volumes, process timings, distribution routes, and incident–response actions. Such data are typically protected by confidentiality agreements and are rarely disclosed at the granularity needed to reproduce full provenance graphs and compliance checkpoints.

Finally, the scenarios that most strongly test the framework—such as delayed contamination detection or recall decisions—cannot be ethically or operationally simulated in live production environments without risk of unnecessary product withdrawal, reputational damage, or regulatory complications.

For these reasons, the study adopts a synthetic but regulation-consistent evaluation strategy. The dataset preserves the structural and temporal properties required for validation (event completeness, transformation links, control-point coverage, delayed detection) while avoiding unlawful disclosure or unsafe experimentation. In this context, synthetic evaluation is not a simplification, but a necessary pre-deployment validation method for health-aware traceability systems [6].

To assess the practical relevance and technical soundness of the proposed veterinary-integrated EPCIS traceability framework, the evaluation addresses three research questions:

**RQ1. Functional correctness:** Can the framework represent veterinary public health controls within EPCIS event streams and propagate risk information across the transformation and aggregation relationships under realistic dairy production scenarios?

**RQ2. Compliance verification capability:** Can mandatory veterinary and hygiene control points (e.g., quality testing and inspections) be verified automatically using structured traceability data, including detection of missing or delayed controls?

**RQ3. Operational and decision-support impact:** To what extent does event-level provenance, enriched with veterinary data, reduce recall scope compared with conventional time-window-based recall strategies under delayed contamination detection?

To answer these questions, three complementary experiments were conducted using synthetic but regulation-consistent and EPCIS-aligned datasets. The first experiment focuses on functional validation, evaluating risk propagation, end-to-end traceability reconstruction, and automated compliance detection under delayed laboratory findings. The second experiment provides a quantitative evaluation of recall-scope reduction enabled by causal trace-forward queries, compared against a baseline time-window recall heuristic. The third experiment (Section 5.4) assesses the system’s implementation robustness and operational performance, examining data governance constraints, interoperability limitations, and stakeholder adoption dynamics under realistic deployment conditions.

Evaluation metrics include: (i) correctness of risk propagation across derivation links, (ii) completeness of reconstructed provenance chains, (iii) detection of missing mandatory control events, (iv) recall scope (number of recalled cases), precision, and recall under competing recall strategies, and (v) indicative integration latency for relational-to-semantic data translation.

This evaluation design enables controlled and reproducible assessment of food-safety relevance, compliance support, and operational feasibility without requiring access to proprietary industrial traceability systems. Measurements were conducted on a standard workstation (Intel i3 10100, 16 GB RAM), and latency refers to per-update translation without full OWL reasoning materialization.

The code for the experiments is fully available online at Git: https://github.com/giannisvassiliou/EPCIS-VET (accessed on 24 April 2026).

### 7.3. Functional Validation Results

To assess the food-safety relevance of the proposed framework, a synthetic validation scenario was executed to emulate delayed contamination discovery, a frequent situation in dairy supply chains where laboratory test results become available only after production and distribution events have already occurred. The experiment focused on verifying whether the framework can support risk propagation, end-to-end traceability, and regulatory compliance verification under realistic operational conditions.

#### 7.3.1. SyntheticDataset Design

The functional validation was conducted using a synthetic but schema-consistent dataset designed to emulate key structural and temporal properties of dairy supply chains. The dataset was generated programmatically and populated with EPCIS-aligned entities representing farms, milk batches, cheese batches, and associated quality and inspection events.

Each milk batch was linked to a source farm through explicit provenance attributes, while cheese batches were deterministically derived from upstream milk batches via transformation relationships. This ensured that complete provenance chains of the form *Farm→ Milk → Cheese* were available for traceability and recall analysis. Zoonosis risk indicators were assigned at the milk-batch level, with most batches labeled as *Low risk* and a controlled subset retrospectively assigned *High* or *Critical* risk levels to simulate delayed laboratory detections.

To support compliance validation, one milk batch was intentionally generated without an associated quality-testing event, representing a procedural violation of mandatory hygiene controls. This controlled omission enabled verification that missing control points could be detected automatically through semantic queries over the integrated traceability data.

The validation workload consisted of 500 incremental batch updates, each ingested into a relational backend and translated into RDF representations through the proposed SQL-to-RDF integration layer. This configuration allowed simultaneous evaluation of functional correctness (risk propagation, traceability completeness, and compliance detection) and operational feasibility under near-real-time update conditions.

#### 7.3.2. Risk Propagation Validation

After ingestion, a subset of milk batches was retrospectively assigned *High* or *Critical* zoonosis risk levels to simulate delayed laboratory findings. Once these risk indicators were introduced, the semantic integration and rule layer materialized quarantine requirements for downstream cheese batches.

As summarized in Table 3, quarantine status was successfully propagated to all downstream batches derived from high-risk milk lots, confirming the correct implementation of post hoc risk propagation across transformation stages.

#### 7.3.3. Traceability Validation

The framework was further evaluated for its ability to support end-to-end traceability across production stages and organizational boundaries. A SPARQL query linking quarantined cheese batches to their originating milk batches and source farms was executed.

The results, reported in Table 4, confirm that complete provenance chains from cheese batches back to source farms were successfully reconstructed, demonstrating the suitability of the semantic representation for recall management and regulatory investigations.

#### 7.3.4. Regulatory Compliance Validation

To assess compliance-checking capabilities, a procedural violation was intentionally introduced by generating a single milk batch without an associated QualityTestEvent. A semantic compliance query was then executed to identify missing mandatory control points.

As shown in Table 5, the framework correctly identified the non-compliant batch, confirming that mandatory quality control procedures can be automatically verified using integrated inspection and traceability data.

##### Operational Implications of Missing Veterinary Control Evidence

In practice, missing mandatory control-point evidence (e.g., absence of a quality-testing event for a milk lot) introduces uncertainty that may trigger precautionary widening of containment actions. To reflect this operational posture, we define an *uncertainty expansion* rule: if any upstream milk lot contributing to a cheese batch lacks required veterinary/quality-control evidence, then the batch is marked as uncertain and is included in an expanded precautionary recall set (e.g., all batches produced on the same processing line within a fixed time window). This rule does not model biological causation; rather, it models regulator- and operator-driven decision logic under incomplete documentation.

##### Effect on Recall Scope (Documentation-Driven Expansion)

Under the uncertainty expansion rule, the intentionally missing QualityTestEvent caused an expansion of the recalled set beyond the causally linked batch, illustrating how complete veterinary control logging can constrain recall scope by preventing uncertainty-driven precautionary actions.

#### 7.3.5. Performance Overhead

Although performance evaluation was not the primary objective, the end-to-end integration latency—from relational data insertion to semantic graph materialization—was measured under a synthetic workload of 500 updates.

The observed performance metrics, summarized in Table 6, indicate millisecond-scale translation latency for the setup. Millisecond-scale integration latency ensures that quarantine and recall decisions can be triggered without operational delay in high-throughput dairy environments.

#### 7.3.6. Summary

Overall, the functional validation demonstrates that the proposed framework can accommodate delayed contamination discovery, dynamically propagate product risk status across transformation stages, support end-to-end traceability queries, and enable automated regulatory compliance checks. These capabilities, validated empirically in Table 3, Table 4, Table 5 and Table 6, are critical for effective food safety management and regulatory response in complex dairy supply chains.

### 7.4. Quantitative Evaluation: Recall Scope Reduction Under Delayed Contamination

#### 7.4.1. Objective

When causal linkage between products is incomplete, dairy recall actions are often implemented using coarse heuristics, such as facility-wide or time-window-based recalls. While conservative, these strategies frequently result in substantial over-withdrawal of unaffected products. This experiment quantifies the reduction in recall scope enabled by event-level provenance (transformation relationships) in the proposed veterinary-integrated traceability framework, compared against a baseline time-window recall policy.

#### 7.4.2. Synthetic Dataset Description

The quantitative evaluation is conducted on a synthetic, process-consistent cheese supply-chain dataset generated using the default configuration of the recall experiment script. The dataset spans 14 production days and includes 15 dairy farms, 560 raw milk lots (40 per day), and 210 cheese batches (15 per day) produced on two parallel processing lines.

Each cheese batch is derived from exactly k=4 distinct upstream milk lots, yielding explicit milk-to-cheese transformation relationships. Milk lots are annotated with representative veterinary and quality attributes, including somatic cell count, antibiotic residue flags, and herd-health indicators. Cheese batches are assigned to processing lines and maturation rooms to support line-based recall heuristics.

Contamination is injected at the cheese-batch level with a prevalence of 3%, and laboratory confirmation is modeled as a delayed detection occurring between 7 and 14 days after production. Each cheese batch is expanded into a variable number of packaged distribution units, with an average of approximately 60 cases per batch, resulting in roughly 12,000–13,000 cases across the dataset. Packaged cases constitute the unit of recall scope in the evaluation.

The dataset is generated deterministically given a fixed random seed and is exported as CSV files encoding milk lots, cheese batches, transformation events, packaged cases, laboratory detections, and recall outcomes.

#### 7.4.3. Recall Strategies

Let a laboratory detection event identify a contaminated cheese batch *b* at time $t_{d}$. Two recall strategies are evaluated.

##### Baseline (Time-Window Recall)

All cheese batches produced on the same processing line as *b* within ±W hours of the production time of *b* are recalled. The recalled set is as follows:

(7) $$S^{\mathrm{base}}\left(b\right)=\left\{\;c|\mathrm{line}\left(c\right)=\mathrm{line}\left(b\right)\;\wedge \;|t\left(c\right)-t\left(b\right)|\leq W\;\right\},$$

where *c* ranges over cheese batches and t(·) denotes batch production time. All packaged cases derived from batches in $S^{\mathrm{base}}\left(b\right)$ are withdrawn.

##### Proposed (Trace-Forward Recall)

Only packaged cases causally derived from the detected batch *b* are recalled:

(8) $$S^{\mathrm{prop}}\left(b\right)=\left\{\;u|\mathrm{parentBatch}\left(u\right)=b\;\right\},$$

where *u* ranges over packaged cases. This corresponds to trace-forward queries over event-level provenance, avoiding reliance on time-window heuristics.

#### 7.4.4. Evaluation Metrics

Let A(b) denote the set of truly affected packaged cases for detected batch *b* (ground truth in the simulation). Let $R^{\mathrm{base}}\left(b\right)$ and $R^{\mathrm{prop}}\left(b\right)$ be the recalled case sets under baseline and proposed strategies, respectively. We report:

##### Recall Scope (Cases)


(9) $${\mathrm{Scope}}^{m}\left(b\right)=\left|R^{m}\left(b\right)\right|,\;m\in \left\{\mathrm{base},\mathrm{prop}\right\}.$$

##### Precision and Recall


(10) $${\mathrm{Precision}}^{m}\left(b\right)=\frac{|R^{m}\left(b\right)\cap A\left(b\right)|}{|R^{m}\left(b\right)|},\;{\mathrm{Recall}}^{m}\left(b\right)=\frac{|R^{m}\left(b\right)\cap A\left(b\right)|}{\left|A(b)\right|}.$$

##### Scope Reduction


(11) $$\mathrm{SR}\left(b\right)=\frac{{\mathrm{Scope}}^{\mathrm{base}}\left(b\right)-{\mathrm{Scope}}^{\mathrm{prop}}\left(b\right)}{{\mathrm{Scope}}^{\mathrm{base}}\left(b\right)}\times 100\%.$$

#### 7.4.5. Results and Interpretation

Across n=9 contamination detection events, the distribution of recall scope, precision, and recall for both strategies is summarized in Table 7. Under the evaluated configuration (e.g., W=24 h), the baseline time-window strategy achieves perfect recall, but low precision due to the inclusion of non-affected batches produced within the recall window.

In contrast, the proposed trace-forward strategy recalls only cases causally derived from the detected batch, yielding perfect precision and substantially reducing recall scope. As shown in Table 7, the median number of recalled cases is reduced from 950 under the baseline strategy to 62 under the proposed strategy, corresponding to a median recall-scope reduction of 93.0%. However, this result reflects idealized conditions with complete and accurate provenance data. As shown in Section 7.4, when cross-contamination, incomplete capture, or detection uncertainty are introduced, purely causal trace-forward recall may provide insufficient safety coverage, and hybrid precautionary strategies may be required. Under realistic uncertainty, hybrid precautionary policies provide more robust safety coverage than pure trace-forward recall.

#### 7.4.6. Notes on Assumptions and Limitations

This evaluation isolates the impact of causal traceability on recall scope under controlled conditions. Real-world deployments may require modeling additional sources of uncertainty, such as imperfect detection, cross-contamination, or incomplete event capture. Nevertheless, the experiment provides a reproducible quantitative KPI—recall-scope reduction—that demonstrates the operational benefit of event-level provenance compared with time-window-based recall heuristics.

### 7.5. Comparison of Recall Policies Under Uncertainty

The baseline evaluation in the previous section demonstrates the benefit of event-level provenance for targeted recall scoping under delayed detection. However, real dairy supply chains operate under additional sources of uncertainty that can weaken purely causal (trace-forward) recall decisions. In particular, (i) microbiological hazards may spread beyond a single batch through shared equipment, processing lines, or maturation environments, and (ii) traceability repositories may exhibit incomplete event capture (e.g., missing transformation/aggregation links). To assess whether the proposed approach remains useful under such non-ideal conditions, we conducted a robustness evaluation that explicitly models cross-contamination, imperfect detection, and incomplete provenance capture.

#### 7.5.1. Experimental Design

We extended the synthetic dataset generator to include two processing lines and multiple maturation rooms, with milk-to-cheese transformation relationships defined for each cheese batch. We then introduced three realism-oriented perturbations:

1. **Cross-contamination:** When a contaminated batch is present, other batches may become contaminated due to exposure. We model exposure along (a) the same processing line within ±E hours, and (b) the same maturation room within ±R days. Exposure-linked contamination occurs probabilistically using parameters $p_{\mathrm{line}}$ and $p_{\mathrm{room}}$.
2. **Incomplete capture (missing links):** With probability $p_{\mathrm{link}\text{\_}\mathrm{miss}}$, provenance links are removed (e.g., missing transformation linkage), representing incomplete event capture or integration gaps.
3. **Detection uncertainty:** Laboratory detection is delayed by a bounded random delay and may fail to detect contaminated batches with probability $p_{\mathrm{fn}}$ (false negatives), representing imperfect testing and reporting uncertainty.

These perturbations intentionally violate the idealized assumptions under which purely causal trace-forward recall is expected to be optimal, thereby enabling a more demanding evaluation of recall policies.

#### 7.5.2. Recall

Strategies For each detection event identifying batch *b*, we evaluate four recall policies:

- **Baseline (time-window):** Recall all batches on the same line as *b* produced within ±W hours of *b*’s production time.
- **Baseline (line + room):** Recall all batches on the same line and in the same maturation room as *b* produced within ±W hours.
- **Trace-forward:** Recall only the detected batch (i.e., causal recall bounded to the detected entity, representing the most conservative interpretation of batch-local provenance in the presence of missing links).
- **Hybrid:** Trace-forward recall plus a precautionary exposure buffer: Batches within the exposure neighborhoods of *b* (line within ±E hours and/or room within ±R days).

The hybrid policy operationalizes a realistic incident-response posture: use event-level provenance for precision, but add limited exposure buffers to protect public health when environmental spread is plausible.

#### 7.5.3. Metrics

Let *A* be the set of truly affected (contaminated) batches under the cross-contamination model, and let $R_{m}$ be the set of recalled batches under strategy *m*. We report:

$${\mathrm{Precision}}_{m}=\frac{|R_{m}\cap A|}{|R_{m}|},\;{\mathrm{Recall}}_{m}=\frac{|R_{m}\cap A|}{\left|A\right|},\;F1_{m}=\frac{2\;{\mathrm{Precision}}_{m}\;{\mathrm{Recall}}_{m}}{{\mathrm{Precision}}_{m}+{\mathrm{Recall}}_{m}},$$

and **scope** as $|R_{m}|$ (number of recalled batches). We aggregate results by computing medians across detection events within each run, and then summarize across stochastic seeds.

#### 7.5.4. Parameter Sweep and Reproducibility

We performed a controlled parameter sweep across 36 configurations, varying key robustness drivers (e.g., missing-link probability and time-window width), and repeated each configuration across 10 stochastic seeds (360 runs total). The experiment produces both (i) a long-form results table for reproducibility and (ii) summary statistics aggregated by configuration and strategy.

#### 7.5.5. Results

Table 8 reports top-line aggregated performance (mean of per-configuration medians) across the evaluated robustness sweep. The results illustrate a clear precision–recall trade-off frontier. The trace-forward strategy achieves perfect precision (1.0) with minimal scope (1 batch), but exhibits low recall under cross-contamination and incomplete capture. In contrast, the hybrid strategy yields the highest recall and the best overall balance (highest F1), at the cost of increased recall scope due to precautionary exposure buffering. Baseline heuristics trade scope for limited gains in recall and can underperform when contamination spreads, and event incompleteness interacts.

From a food-safety governance perspective, the hybrid policy provides a balanced compromise between precautionary public-health protection and avoidance of unnecessary product destruction.

Overall, this robustness study demonstrates that (i) purely causal recall maximizes precision but can under-recall in the presence of environmental spread and incomplete capture, and (ii) a hybrid policy combining event-level provenance with bounded exposure buffers can substantially improve public-health protection (recall) while remaining structured and reproducible. In food safety, under-recall and over-recall are not equivalent. Failing to withdraw contaminated product poses direct public health risks. Over-withdrawal causes economic losses, but these are recoverable. Under this framing, recall is the primary decision-relevant metric, and precision - though reported for methodological completeness—is a secondary consideration for recall policy selection. The hybrid strategy achieves a recall of 0.247, more than four times that of trace-forward (0.055). This improvement in public health protection justifies the larger recall scope. Crucially, unlike time-window heuristics, the hybrid scope remains structured, auditable, and parameterically adjustable—making it both safer and more defensible to regulators than current practice.

##### Precision–Recall Trade-Off Under Uncertainty

Under ideal conditions with complete and accurate provenance data, trace-forward recall based on causal linkage provides high precision, as only directly affected batches are included. However, when more realistic conditions are introduced—such as cross-contamination pathways, missing traceability links, or delayed and uncertain detection signals—the recall of such approaches may degrade, as not all affected products can be identified through the recorded provenance graph.

In these cases, broader precautionary strategies that combine causal traceability with risk-based buffers or hybrid decision rules may provide more robust safety coverage, albeit at the cost of increased recall scope. This highlights a fundamental trade-off between recall precision and safety coverage, and suggests that event-level traceability should be used as a component within a broader decision-support framework rather than as a standalone mechanism.

### 7.6. Summary of Recall-Policy Comparison

The evaluation shows that provenance-based recall achieves substantial scope reduction under ideal conditions, but its advantage diminishes under uncertainty. Hybrid policies combining causal traceability with exposure buffers provide more robust safety coverage, highlighting a trade-off between precision and recall that must be considered in operational settings.

#### Contribution of System Components

The evaluation results can be interpreted in an ablation-style manner. A coarse time-window baseline results in a large recall scope due to a lack of causal specificity. Introducing provenance-based traceability significantly reduces recall scope by enabling precise identification of affected batches. The addition of veterinary public health data does not further reduce recall scope under ideal conditions, but contributes to improved robustness under uncertainty by supporting risk assessment, validation of detection signals, and the application of hybrid precautionary strategies.

## 8. Discussion

Integrating veterinary controls into EPCIS-based traceability strengthens food-safety information flows through direct linkage of animal-health and quality-control data to event streams. This enables targeted recalls at the item- or lot-level rather than broad batch-level withdrawals, while enhancing transparency across farms, processors, distributors, and regulatory authorities. The framework reduces fragmentation between the veterinary and supply-chain systems and, by relying on standard EPCIS constructs, scales naturally to other dairy products and animal-origin supply chains.

### 8.1. Implications for Dairy Food Safety and Recall Management

In dairy production, microbiological hazards such as Listeria monocytogenes are frequently detected after transformation and distribution events, particularly during maturation-stage testing. Under conventional document-based traceability, delayed confirmation often leads to time-window or facility-wide recalls, resulting in substantial over-withdrawal of unaffected products. The event-level provenance model demonstrated in this study enables recall scoping limited to causally linked batches and distribution units. In the synthetic evaluation, median recall scope was reduced by 93% in ideal conditions, while maintaining full recall sensitivity, indicating significant potential for minimizing unnecessary product withdrawal and associated food waste.

Beyond recall scoping, embedding veterinary and hygiene control evidence directly within traceability events strengthens HACCP verification. Mandatory reception checks, inspection outcomes, and laboratory confirmations become queryable, machine-verifiable records rather than parallel documents. This supports faster regulator-ready auditing and reduces the need for manual cross-referencing between farm-level veterinary systems and processing records. For medium-scale dairy plants, such structured evidence capture may reduce incident response time and improve decision consistency during contamination events.

There are still several challenges in putting this system into practice. Veterinary health data and farm-level information are sensitive under GDPR rules; thus, clear data protection and governance procedures are required.

Standard terms and measurement units for veterinary diagnoses must be harmonized across regions to ensure consistency. Adoption can also be difficult because farmers, veterinarians, processors, and authorities differ in their level of digital readiness, motivation, and understanding of the benefits.

Technical integration is another challenge, as many existing systems are outdated and cannot easily share EPCIS event data. These issues highlight the need for strong governance, better system interoperability, and incentive structures that fit both legal requirements and everyday operational needs.

### 8.2. Deployment Scenario in Operational Dairy Plants

To evaluate practical applicability from a dairy food-safety perspective, this subsection describes how the proposed veterinary-aware traceability framework could be implemented in a medium-sized European cheese plant operating under routine HACCP and EU hygiene requirements.

#### 8.2.1. Integration with Existing Production and Quality Systems

Most dairy plants already use Enterprise Resource Planning (ERP), Manufacturing Execution Systems (MESs), and Laboratory Information Management Systems (LIMSs) to manage production orders, batch records, and microbiological testing. The proposed framework does not replace these systems; rather, it structures their existing outputs into standardized traceability events.

Milk reception records, batch transformation logs, laboratory results (e.g., somatic cell counts, antibiotic screening, maturation-stage microbiological tests), and shipment data are already digitally captured in most facilities. These records can be expressed as structured traceability events linked to specific milk lots and cheese batches. Veterinary inspection summaries and laboratory confirmations (e.g., Listeria monocytogenes detection) can be attached to these events as structured extensions, allowing them to become part of the searchable food-safety record without altering established operational workflows.

From a HACCP standpoint, this means that mandatory reception controls, verification testing, and release decisions are no longer stored solely as parallel documents but become integrated elements of the traceability chain. During contamination events, this structured linkage enables faster identification of affected products and clearer documentation of control measures.

#### 8.2.2. Use of Standardized Identifiers for Recall Precision

The deployment leverages existing GS1 identification practices commonly used in dairy logistics, including product codes, location identifiers, and batch/lot numbers. Because these identifiers are already present in barcoding or RFID systems, the framework builds upon familiar infrastructure rather than introducing proprietary schemes.

This continuity is important for recall management. By maintaining consistent identifiers from milk reception through cheese maturation and distribution, the system supports precise trace-forward queries during incident investigations. Instead of relying on time-window heuristics, recall decisions can be bounded to causally linked batches and distribution units, reducing unnecessary withdrawal of unaffected products.

#### 8.2.3. Operational Data Volume and Feasibility

For a representative dairy plant producing approximately 40–60 milk lots and 15–25 cheese batches per day, total traceability event generation (including reception, transformation, inspection, testing, and shipping records) would typically remain below one million events per year. Such volumes are well within the capacity of conventional relational database systems already deployed in food manufacturing environments.

Importantly, routine operational queries—such as stock visibility, batch status, or shipment tracking—remain lightweight and compatible with existing database performance expectations. The structured addition of veterinary and hygiene control information, therefore, enhances food-safety decision support without imposing unrealistic computational requirements.

#### 8.2.4. Cross-Organizational Exchange and Regulatory Reporting

In practical dairy supply chains, contamination investigations frequently require coordination between farms, processors, laboratories, and competent authorities. The structured event-based design supports controlled data sharing between actors while preserving confidentiality.

Authorized stakeholders can access trace-back and trace-forward information necessary for outbreak response, while sensitive veterinary details remain restricted according to role-based permissions. This enables regulator-ready reporting and structured evidence provision during audits or recall coordination, reducing reliance on manual document collation across separate systems.

#### 8.2.5. Operational Readiness

Because the framework builds on established traceability standards and conventional database technologies, it represents an incremental enhancement rather than a disruptive transformation. Its primary contribution lies in integrating veterinary and hygiene evidence directly into traceability records, thereby strengthening recall precision, auditability, and food-safety responsiveness in operational dairy plants.

### 8.3. Scalability and Multi-Plant Deployment Considerations

Beyond single-plant implementation, the framework can support coordinated deployment across multi-facility dairy enterprises or regional supply chains.

#### 8.3.1. Scalable Deployment for Multi-Site Dairy Operations

In larger dairy groups operating multiple production sites, traceability records may be maintained locally while selected events are shared across facilities or with central oversight bodies. Because the proposed approach separates routine operational queries from risk-assessment and compliance validation tasks, high-frequency production data remain efficiently handled, while recall investigations activate more advanced trace-forward analyses only when required.

This design ensures that increasing production throughput does not proportionally increase analytical overhead, preserving system responsiveness during routine operations while supporting rapid escalation during contamination events.

#### 8.3.2. Interoperability Across Actors

The structured event format supports controlled information exchange between farms, processors, laboratories, and authorities. This is particularly relevant for zoonotic hazard management, where upstream veterinary findings may need to be linked to downstream food products. The ability to share selected traceability elements without centralizing all data aligns with emerging European data-sharing initiatives while respecting data sovereignty and confidentiality constraints.

#### 8.3.3. Performance and Scalability Limitations

As production volume increases, event records scale proportionally with batch throughput. Because recall scoping and compliance verification are triggered selectively (e.g., upon detection of non-compliance or pathogen presence), computational demand remains bounded during normal operation. The reported millisecond latency reflects event translation and query execution in a single-node environment, not full reasoning or distributed processing.

The performance evaluation presented in this work is based on synthetic, regulation-consistent datasets and limited-scale workloads. The reported latency results reflect translation and query operations under these controlled conditions and do not include full OWL reasoning materialization or large-scale distributed execution. As such, the results should be interpreted as indicative of feasibility rather than as evidence of enterprise-scale performance. Comprehensive evaluation under realistic data volumes, multi-actor deployments, and full semantic processing remains an important direction for future work.

### 8.4. Future Evaluation Pathways

While the present study demonstrates recall-scope reduction and compliance verification under controlled scenarios, future work should evaluate the framework in real dairy environments. Relevant performance indicators include the following:

Reduction in recall scope compared with historical time-window recalls;

Time required to assemble regulator-ready audit documentation;

Reduction in manual cross-referencing between veterinary and production records;

Impact on food waste and economic loss during contamination events.

Pilot implementation in operational dairy plants would enable quantitative assessment of these food-safety outcomes under real production conditions.

### 8.5. Data Governance and Privacy Considerations

Veterinary records, farm identifiers, and laboratory results may constitute sensitive data under GDPR, particularly when linked to identifiable holdings or individuals. Any operational deployment, therefore, requires clearly defined governance structures specifying data controllers, legal basis for processing, retention policies, and audit mechanisms.

The proposed event-based design supports privacy-preserving configurations by limiting shared traceability records to essential identifiers and control outcomes, while detailed veterinary documentation can remain in restricted repositories. Role-based access control ensures that processors access food-safety-relevant information necessary for product release decisions, while extended veterinary attributes are available only to competent authorities.

By embedding food-safety-relevant summaries within structured traceability events, the framework enhances outbreak response and compliance verification without mandating indiscriminate sharing of sensitive veterinary data.

Overall, the proposed veterinary-integrated traceability framework extends EPCIS-based systems beyond logistical transparency by embedding structured food-safety controls and regulatory checkpoints directly within event-level provenance. In dairy production environments characterized by delayed microbiological confirmation and multi-stage transformations, this integration supports more responsive outbreak containment, narrower recall scoping, and auditable compliance verification. The framework, therefore, primarily contributes to improved dairy food-safety responsiveness, reduced unnecessary product withdrawal, and strengthened regulatory compliance.

### 8.6. Governance-by-Design for Cross-Organizational Veterinary Traceability

To strengthen the deployability of the proposed framework, governance requirements must be specified at the same level of precision as the data model and system architecture. In the original version of this manuscript, GDPR compatibility was discussed mainly at the principle level. In practice, however, lawful cross-organizational veterinary traceability requires explicit decisions regarding which data elements are shared, which actors may access them, for what purpose they are processed, how long they are retained, and under what authority pseudonymized identities may be resolved. For this reason, the framework should be understood not as a single undifferentiated data space, but as a layered governance design in which only the minimum necessary traceability evidence is exposed in the shared EPCIS layer, while sensitive identifying information remains under restricted control.

The governance principle adopted here is selective disclosure with controlled re-identification. Routine supply-chain traceability and recall coordination require access to batch identifiers, transformation links, product status, and selected coded veterinary or inspection outcomes. They do not require unrestricted access to directly identifying farm-owner or full veterinary-record information. Accordingly, the shared traceability layer is designed to store only those attributes needed for provenance, compliance verification, and risk signaling, while direct identifiers and detailed veterinary records remain outside routine inter-organizational exchange and are referenced, where necessary, through pseudonymized or coded links.

This separation supports data minimization and purpose limitation. Processors, distributors, and other operational actors may need access to batch-level provenance, release status, and restricted compliance indicators in order to execute trace-back, trace-forward, hold, and recall workflows. Competent authorities, by contrast, may require broader access in the context of official controls, outbreak investigation, or enforcement. The proposed framework, therefore, assumes role-based access control, in which visibility is determined by actor role and processing purpose rather than by uniform access to all traceability data.

A second governance requirement concerns lawful identity resolution. In cross-organizational settings, pseudonymization is only effective if re-identification is tightly controlled. For that reason, the framework assumes that the mapping between pseudonymized farm references and directly identifying information is held by a designated trusted controller or competent authority, and that re-identification is performed only under auditable and legally justified procedures. This avoids routine disclosure of sensitive farm-linked or owner-linked information while preserving the possibility of targeted investigation when required for food-safety intervention.

A third requirement concerns retention. Different data categories have different retention justifications and operational lifetimes. Batch provenance and recall-relevant event histories may need to remain available for traceability, audit, and regulatory review, whereas directly identifying personal data should be retained only for the minimum period necessary under the applicable legal and sectoral rules. The framework, therefore, separates operational retention of traceability evidence from restricted retention of identifying or case-level veterinary records.

Table 9 summarizes the governance design choices assumed by the proposed framework. Its purpose is not to prescribe a single legally binding implementation for all jurisdictions, but to make explicit the engineering assumptions required to render veterinary-aware traceability lawful, auditable, and deployable across multiple actors.

The matrix clarifies that the proposed framework does not require unrestricted sharing of sensitive veterinary or personal data in order to support traceability and recall optimization. Instead, it relies on a layered architecture in which the shared event layer carries minimally necessary, machine-actionable evidence, while direct identity resolution remains exceptional, restricted, and auditable. This distinction is essential for translating high-level privacy principles into an implementation model that can plausibly operate across farms, processors, laboratories, and competent authorities.

### 8.7. Limitations and Practical Considerations

While the proposed framework provides a structured and standards-aligned approach for integrating veterinary public health data into EPCIS-based traceability systems, several limitations should be acknowledged in order to provide a balanced interpretation of its contribution. The paper already clarifies that it adopts a design-oriented and pre-deployment validation perspective, rather than reporting an operational industrial deployment, and that different components of the framework are supported by different levels of evidence.

First, the evaluation relies on synthetic, regulation-consistent datasets rather than real-world deployment data. This choice enables controlled validation of provenance tracking, risk propagation, and recall-scope behavior, but it does not fully capture the heterogeneity, incompleteness, latency, and noise that characterize operational dairy traceability environments. Consequently, the quantitative results should be interpreted as indicative of the framework’s potential under controlled assumptions, rather than as direct evidence of field performance.

Second, the proposed approach introduces non-trivial integration complexity. The framework assumes that veterinary records, laboratory information, farm-level data, and production traceability events can be aligned through shared identifiers, schema mappings, and EPCIS-compatible extensions. In practice, this may require substantial data engineering effort, semantic harmonization, and cross-organizational coordination, especially where legacy systems do not natively support structured export of veterinary public health information.

Third, the hybrid SQL–RDF/OWL architecture may impose additional computational and operational overhead compared with conventional EPCIS deployments. Although the design separates routine operational queries from selective semantic processing, real deployments may still face performance trade-offs when provenance graphs become large, when rule evaluation becomes frequent, or when near-real-time compliance checks are required. For this reason, the architecture should be understood as a flexible design pattern rather than a universally low-cost implementation choice.

Fourth, the effectiveness of the framework depends heavily on data availability and data quality. Delayed laboratory confirmation, missing inspection records, inconsistent reporting practices, or incomplete linkage between upstream and downstream entities may reduce the accuracy of automated risk propagation and compliance verification. In such cases, the framework remains useful as a traceability support mechanism, but its outputs may still require human review and regulatory interpretation.

Fifth, the operational adoption of the framework may be constrained by organizational and resource-related factors. Its implementation requires cooperation among farms, processors, laboratories, inspectors, and competent authorities, all of whom may differ in digital maturity, interoperability readiness, and willingness to share data. These challenges may be particularly significant for small and medium-sized enterprises, for which the additional infrastructure, integration, and governance requirements may be perceived as costly relative to immediate operational benefits.

Sixth, although the framework incorporates privacy-aware mechanisms such as pseudonymisation, attribute minimisation, and layered access control, privacy-preserving deployment remains a practical challenge. In particular, there is an inherent trade-off between protecting sensitive farm- and veterinary-related information and enabling sufficiently rich cross-organizational analytics for risk assessment, compliance auditing, and outbreak investigation.

Finally, the present work is domain-specific. The framework is developed around dairy supply chains, and especially cheese-production settings characterized by multi-stage transformations, delayed microbiological results, and mandatory veterinary control points. While the general design principles may be transferable to other agri-food sectors, the specific mappings, control predicates, and risk logic would likely require adaptation before broader application.

Taken together, these limitations do not reduce the conceptual value of the proposed framework; rather, they define the boundaries within which its current contribution should be interpreted. The present study should therefore be seen as a standards-aligned, pre-deployment foundation for veterinary-aware digital traceability, with future work needed to evaluate implementation cost, scalability, usability, and regulatory effectiveness in operational settings.

## 9. Conclusions

Dairy products remain particularly sensitive to zoonotic hazards and hygiene failures, requiring traceability systems that support rapid, precise, and evidence-based food-safety decisions. This study presented a veterinary-integrated EPCIS framework that embeds herd-health context, milk quality indicators, inspection checkpoints, and laboratory findings directly into event-level dairy traceability records. By linking animal-health intelligence with product transformation and aggregation events, the system enables health-aware provenance tracking across milk-to-cheese supply chains.

The proposed Health-Aware Traceability Model formalizes risk propagation and compliance verification over event-level provenance graphs, allowing delayed contamination signals—such as *Listeria monocytogenes* detection during maturation—to be systematically propagated to all causally derived products. Empirical evaluation using regulation-consistent datasets demonstrated correct risk inheritance across transformation stages, automated identification of missing mandatory control events, and complete end-to-end provenance reconstruction. Quantitative recall analysis showed that event-level trace-forward scoping reduced recall scope under controlled conditions compared with time-window-based recall strategies while maintaining full recall accuracy, but further validation in real-world deployments is required. Even under robustness scenarios including cross-contamination and incomplete event capture, hybrid policies combining causal traceability with bounded exposure buffers provided structured and defensible recall boundaries. These findings highlight the operational relevance of integrating veterinary public health data directly into digital traceability infrastructures.

Beyond technical interoperability, the framework contributes to food-safety governance by embedding compliance evidence within standardized EPCIS event streams aligned with EU Regulations 852/2004 and 853/2004. This integration enhances auditability, reduces reliance on parallel documentation systems, and supports regulator-ready reporting while respecting data-protection principles.

Overall, the proposed approach shifts digital traceability in dairy supply chains from passive record-keeping toward active food-safety decision support. By enabling targeted recalls, reducing unnecessary product withdrawal and food waste, and strengthening the linkage between animal health and processed food outcomes, the framework offers a scalable foundation for risk-resilient and transparent dairy supply chains. Future work will focus on pilot deployment in operational dairy environments and extension to other animal-origin food sectors.

## Figures and Tables

**Table 1:** Comparison between representative EPCIS deployment and the proposed veterinary-integrated system.

Feature	Kassahun et al. [21]	Typical EPCIS	This Work
EPCIS event capture	Yes	Yes	Yes
Cloud-based architecture	Yes	Yes	Yes
Structured veterinary data encoding	No	No	Yes
Zoonotic risk classification	No	No	Yes
Automated compliance verification	No	No	Yes
Quantitative recall-scope evaluation	No	No	Yes

**Table 2:** Expanded mapping of framework concepts to EPCIS and schema elements.

Framework Concept	EPCIS Construct	Schema Elements/Examples
Milk lot/raw material	ObjectEvent + ILMD	object_event.ilmd_json (SCC, fat, antibiotics), event_epc_list
Milk → cheese transformation	TransformationEvent	transformation_input_epc (milk lot), transformation_output_epc (cheese batch)
Veterinary inspection at a farm	ObjectEvent (inspection)	biz_step: veterinary_inspection, extensions_json (result, findings)
Herd health status	Master data (parties)	parties.attrs (brucellosis free, vaccination dates)
Zoonosis risk classification	Disposition + lot metadata	lots.attrs (riskAssessment), disposition: quarantine
Environmental conditions	SensorElement/SensorReport	temperature/humidity values with timestamps
Regulatory batch release	Commissioning event	biz_step: commissioning, lots.certificate_url

**Table 3:** Risk propagation validation results.

Metric	Result
High/Critical-risk milk batches	Present
Quarantine triples materialized	13
Risk propagation check	PASS

**Table 4:** Traceability query results for high-risk products.

Metric	Result
Traceability query rows returned	13
Provenance chain reconstructed	Cheese → Milk → Farm
Traceability validation	PASS

**Table 5:** Regulatory compliance checking results.

Metric	Result
Milk batches missing quality test	1
Expected missing tests	1
Compliance detection check	PASS

**Table 6:** End-to-end integration performance.

Metric	Value
Number of updates	500
Median translation latency	1.06 ms
95th percentile latency	2.19 ms

**Table 7:** Recall-scope comparison between baseline (time-window) and proposed (trace-forward) strategies across contamination detection events (n=9). Values reported as median (interquartile range).

Metric	Baseline	Proposed	Improvement
Recalled cases (Scope)	950 (912–990)	62 (53–65)	–
Precision	0.07	1.00	+93 pp
Recall	1.00	1.00	0
Scope reduction (%)	–	–	93.0 (92.2–93.3)

**Table 8:** Robustness evaluation: top-line performance (mean of median metrics) across 36 configurations and 10 seeds. Scope is measured as the number of recalled batches.

Strategy	Precision	Recall	F1	Scope
Baseline (time window)	0.187	0.118	0.137	11.500
Baseline (line + room)	0.349	0.076	0.120	4.250
Trace-forward	1.000	0.055	0.104	1.000
Hybrid (trace + exposure)	0.145	0.247	0.183	31.333

**Table 9:** Illustrativedata-governance matrix for cross-organizational veterinary-aware traceability.

Data Element	Shared in EPCIS Layer	Main Actors	Purpose	Lawful Basis/Processing Context	Access Rights	Retention	Pseudonym-Resolution Authority
Batch identifiers, product lot references, transformation and aggregation events	Yes	Processor, distributor, competent authority	Traceability, recall, audit	Operational traceability and compliance processing	Broad operational access	According to traceability and audit requirements	Not applicable
Milk-quality test result linked to lot	Yes (coded or referenced)	Processor, laboratory, competent authority	Release control, compliance verification, investigation	Food-safety and compliance processing	Restricted operational access; authority access when required	Compliance retention period	Competent authority or designated controller if re-linking is required
Herd-health status indicator/coded risk flag	Yes (coded form)	Processor, competent authority	Risk signaling, precautionary decision support	Regulatory or food-safety processing context	Role-based restricted access	Veterinary compliance retention period	Competent authority only
Detailed veterinary record	No (reference only)	Veterinarian, competent authority	Official control, investigation, case review	Restricted veterinary public-authority processing	No routine supply chain access	According to sectoral legal rules	Competent authority only
Farm identifier	Pseudony- mized only	Processor, competent authority	Controlled linkage of provenance to source actor	Minimized identification for traceability and investigation	Very limited, role-based access	Minimum necessary period	Designated trusted controller or competent authority
Owner personal data	No in routine shared layer	Competent authority only	Enforcement or legally required follow-up	Separate controlled processing under applicable law	No general supply-chain access	Minimum legally necessary period	Competent authority only
Inspection outcome/compliance status	Yes	Processor, auditor, competent authority	Compliance evidence, release/hold decisions	Compliance and official-control processing	Role-based access	Audit - compliance retention period	Not usually required

## Data Availability

The original contributions presented in this study are included in the article. Further inquiries can be directed to the corresponding author.

## Abbreviations

The following abbreviations are used in this manuscript:

EPCIS	Electronic Product Code Information Services (GS1 standard for capturing and sharing supply chain event data—“what, when, where, why”).
CBV	Common Business Vocabulary (Standardized business terms used with EPCIS events, e.g., bizStep, disposition).
Event Types	Structured EPCIS record formats describing actions or states in a product lifecycle.
ObjectEvent	EPCIS event type tracking an item at a specific place and time.
TransformationEvent	EPCIS event type capturing transformation of inputs into outputs (e.g., milk into cheese).
AggregationEvent	EPCIS event type grouping multiple items (e.g., cheese wheels into a pallet).
TransactionEvent	EPCIS event type linking items to business transactions (e.g., purchase orders).
SensorEvent	EPCIS event type associating environmental sensor data (e.g., temperature, humidity) with items or batches.
ILMD	Instance/Lot Master Data (Additional descriptive attributes associated with a product during an EPCIS event, e.g., fat content, lab results).
EPC	Electronic Product Code (Globally unique identifier for a specific item or batch, enabling end-to-end traceability).
GLN	Global Location Number (GS1 identifier for uniquely identifying locations or legal entities where events occur).
Parties Table	Master data structure storing information about supply chain actors (e.g., farms, processors, inspectors).
Lots Table	Batch metadata structure representing raw material or product lots, including risk or compliance attributes.
Sensor Report	Structured environmental condition record (e.g., cold-chain temperature, humidity) captured via IoT and linked to EPCIS events.
Data Governance	Policies and practices defining how data is collected, stored, shared, protected, and retained.
Pseudonymisation	Privacy technique replacing identifying data with coded values while allowing controlled re-identification.
GDPR	General Data Protection Regulation (EU regulation governing the processing and protection of personal data).
EU Regulation 852/2004	EU regulation establishing general food hygiene, traceability, and hazard control requirements.
EU Regulation 853/2004	EU regulation establishing specific hygiene rules for food of animal origin, including milk and dairy.

## Appendix A. Implementation-Ready Mapping of Veterinary Concepts to EPCIS

This section provides concrete, implementation-ready examples illustrating how veterinary public health information is encoded within the EPCIS data model. The examples are aligned with EPCIS 2.0 and the GS1 Common Business Vocabulary (CBV) and are intended to support reproducibility and system implementation.

### Appendix A.1. Milk Quality Indicators via Instance/Lot Master Data (ILMD)

Milk quality indicators (e.g., somatic cell count, antibiotic residue screening, and compositional parameters) are stored as Instance/Lot Master Data (ILMD) associated with EPCIS Object Events. The EPCIS event references the lot or batch identifier, while the detailed measurements are included in a structured ilmd_json payload.

The corresponding EPCIS Object Event references the ILMD entry:

### Appendix A.2. Herd Health and Vaccination Status via Master Data

Herd health status and vaccination records are maintained at the supplier (farm) level using EPCIS master data attributes (e.g., parties.attrs). These attributes are linked to incoming milk lots through standard EPCIS party-to-event associations.

### Appendix A.3. Zoonosis Risk Indicators and Batch-Level Classification

Zoonosis-related parameters and batch risk classification are recorded at the lot level using structured attributes (e.g., lots.attrs). These attributes reference laboratory tests and pathogen-specific results and may trigger operational state changes via EPCIS dispositions.

In the case of risk escalation, the EPCIS disposition reflects the updated status of the affected lot or batch:

### Appendix A.4. Veterinary and Hygiene Inspection Events

Veterinary and hygiene inspections conducted at critical control points (e.g., farm audits, milk reception, pre-maturation checks) are represented as EPCIS Object Events or Transformation Events enriched with structured inspection metadata.

### Appendix A.5. Environmental Monitoring via EPCIS Sensor Elements

Time-dependent environmental measurements relevant to food safety (e.g., temperature and humidity during cold-chain handling) are integrated using EPCIS sensor elements and sensor reports.

### Appendix A.6. Implementation of Regulatory Requirements in EPCIS

This section details how mandatory hygiene requirements derived from EU Regulations 852/2004 and 853/2004 are mapped to EPCIS business processes, business steps, and dispositions.

### Appendix A.7. Critical Control Points and Business Steps

Key control points in cheese production are represented using CBV-compliant biz_step values:

### Appendix A.8. Non-Compliance Representation and Corrective Actions

Compliance outcomes are encoded using EPCIS dispositions, supplemented with structured metadata describing the nature of the non-compliance and the associated corrective action.

### Appendix A.9. Certification and Laboratory Documentation References

Certification and laboratory documentation are referenced at the lot level using persistent URLs or identifiers. EPCIS events link to the certified lot without embedding the documents themselves.

These examples illustrate how veterinary and regulatory controls can be implemented reproducibly within the EPCIS data model while preserving interoperability, auditability, and regulatory compliance.

## Appendix B. System Ontology

The developed ontology provides a formal semantic model for a cheese supply-chain traceability system integrating EPCIS events, dairy processing operations, and veterinary health controls. Its primary objective is to ensure end-to-end transparency, regulatory compliance, and risk-based decision support from milk collection through processing, storage, and final distribution. The ontology defines the core entities, relationships, and attributes required to standardize data exchange and enable semantic interoperability among farms, processors, laboratories, inspectors, and regulatory authorities.

The ontology scope includes farm-level herd health status, milk reception and transformation processes, critical control points and inspection workflows, batch- and item-level traceability, EPCIS-compatible event modeling, quality and laboratory test results, and cold-chain and environmental monitoring. Structurally, it is organized into four interrelated components: core product and process classes, actor and location concepts, an event-based traceability layer, and veterinary and regulatory control concepts.

Core product classes are modeled under the superclass *FoodProduct* and include *MilkBatch*, *CheeseBatch*, *CheeseWheel*, and *RawMaterial*. Actors and locations comprise *Farm*, *DairyPlant*, *Warehouse*, *Distributor*, *Inspector*, *Laboratory*, and *RegulatoryAuthority*. Traceability is driven by EPCIS-aligned event classes, with *TraceEvent* as a superclass extended by *MilkReceptionEvent*, *CheeseTransformationEvent*, *AggregationEvent*, *SensorEvent*, *QualityTestEvent*, *VeterinaryInspectionEvent*, *RiskAssessmentEvent*, and *CommissioningEvent*. The veterinary and health domain includes *HerdHealthStatus*, *DiseaseStatus*, *ZoonosisRisk*, *InspectionResult*, *HealthCertificate*, and *CriticalControlPoint*.

Object properties capture material flow, responsibility, and regulatory relationships across the supply chain. Milk batches are linked to farms via *sourcedFrom*, cheese batches are connected to their inputs through *derivedFromMilk*, and transformation events relate input and output products using *hasInputProduct* and *hasOutputProduct*. Events are associated with products (*hasProduct*), actors (*performedBy*), and locations (*occursAt*). Quality, inspection, and risk information is represented through *hasQualityTest*, *hasInspectionResult*, *hasZoonosisRisk*, *hasHerdHealthStatus*, *hasValidCertificate*, and *inspectedControlPoint*.

Datatype properties describe measurable and regulatory attributes, including somatic cell count, fat and protein content, antibiotic residue status, listeria and salmonella test outcomes, event timestamps, temperature and humidity values, registration and certificate identifiers, quarantine requirements, and inspection pass/fail status. These attributes support both descriptive analytics and automated compliance verification.

The ontology enables automated reasoning through explicit axioms. Each *MilkBatch* must be associated with at least one quality test. Elevated zoonosis risk levels propagate quarantine requirements to all derived *CheeseBatch* instances. Cheese batches cannot be commissioned without a valid health certificate, and any failed veterinary inspection automatically triggers a mandatory risk assessment. These rules support proactive hazard containment and regulatory enforcement.

The ontology is compatible with EPCIS 2.0 and supports event-based data capture, ILMD enrichment for quality attributes, GS1 identification keys (GTIN, GLN, GRAI), and regulatory traceability reporting. It is deployed within a hybrid architecture in which a relational SQL database acts as the transactional source of truth, while a translation service converts database notifications into RDF triples stored in a semantic triple store. This approach enables ACID-compliant operations alongside advanced semantic querying and OWL-based reasoning through a unified API layer.

## Appendix C. SQL-to-RDF Translation Layer

Although the conceptual framework of the proposed system is grounded in RDF/OWL semantics to enable reasoning and semantic interoperability, its practical implementation adopts a hybrid architecture that balances operational performance with standards compliance. In this approach, a relational SQL database serves as the primary operational backend, while all relevant data modifications are automatically translated into RDF triples according to the RDFS schema defined in Section 5. This translation layer bridges high-throughput transactional storage with semantic querying capabilities, enabling both efficient operations and interoperable data exchange.

The architectural choice is motivated by operational, performance, and adoption constraints. Existing dairy traceability platforms, ERP systems, and EPCIS repositories are predominantly based on relational databases such as PostgreSQL, MySQL, and Oracle, making a full migration to native triple stores impractical. Transactional supply-chain workloads require frequent writes with ACID guarantees and low latency, which SQL databases handle efficiently, whereas triple stores typically incur higher write costs. In addition, SQL expertise is widely available among supply-chain IT teams, while SPARQL and triple-store administration remain specialized skills. Despite this, semantic interoperability remains a core requirement, necessitating RDF-based interfaces for regulatory reporting, cross-organizational querying, and integration with Linked Data ecosystems. The translation layer ensures that all relational updates are consistently reflected in the RDF representation without compromising operational efficiency.

The relational schema is designed to closely mirror the ontology structure and facilitate deterministic mapping to RDF. Core tables represent product batches, actors, events, and environmental measurements. Batch entities such as milk and cheese are stored in a unified lots table using a discriminator column to distinguish product types, while semi-structured quality, veterinary, and risk attributes are stored in JSONB fields. Actors, including farms and processing facilities, are represented in a parties table, also enriched with JSONB attributes for herd health and vaccination records. EPCIS-compliant event data are captured in an object_events table, which stores timestamps, business steps, dispositions, and extension attributes, while environmental monitoring data are maintained in a dedicated sensor_reports table.

**Listing A1.** Core relational schema for traceability data.

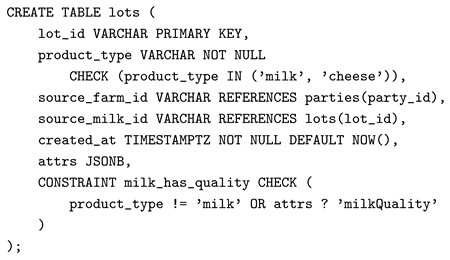

All INSERT and UPDATE operations on relevant tables trigger real-time translation to RDF triples. This mechanism is implemented using PostgreSQL’s LISTEN/NOTIFY facility in combination with a dedicated translation service. Database triggers capture row-level changes, serialize the modified data as JSON payloads, and emit notifications on a synchronization channel consumed by the translation service.

**Listing A2.** PostgreSQL trigger for RDF synchronization.

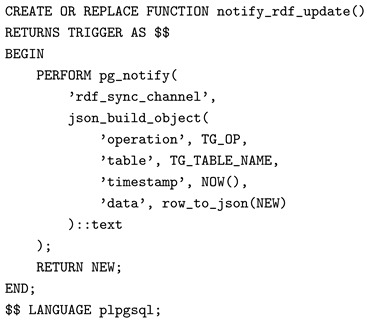

The translation service, implemented in Python v.3.10, listens for database notifications and converts relational rows into RDF graphs using deterministic mapping rules. Primary keys are transformed into subject URIs, discriminator columns determine RDF classes, foreign keys become object properties, scalar columns map to datatype properties, and nested JSONB structures are represented using blank nodes for intermediate ontology classes.

**Listing A3.** RDF translation service (conceptual).

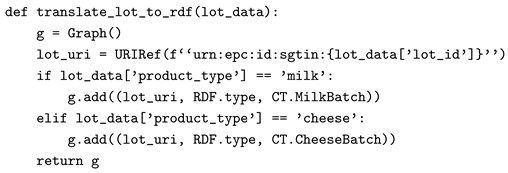

Once RDF triples are materialized in the graph database, the system supports standard SPARQL queries for regulatory reporting, outbreak investigation, and cross-organizational analytics. Queries can trace high-risk cheese batches back to their source farms, verify compliance with mandatory quality testing rules, and aggregate herd health indicators across suppliers. These capabilities enable semantic reasoning and graph traversal that are impractical to express efficiently in pure SQL.

Deployment is envisioned as a phased process, beginning with batch-based RDF export for validation, followed by trigger-driven synchronization, horizontal scaling in production, and eventual activation of OWL reasoning and SHACL-based compliance checking. Overall, the hybrid architecture preserves SQL as the authoritative source of truth while exposing a standards-compliant RDF interface, enabling high-throughput operations, semantic interoperability, incremental adoption, and future extensibility for advanced reasoning and federated querying.

## Appendix D. RDF Schema for the System Ontology

RDF Schema for the System Ontology The RDF Schema defines the formal structure of the proposed ontology by specifying the core classes, object properties and datatype properties required to model traceability, processing activities and veterinary controls across the cheese supply chain. It provides a machine-interpretable foundation that ensures semantic consistency between heterogeneous information systems, supports automated reasoning for risk assessment and certification validation, and enables reliable backward and forward traceability from milk collection to finished product distribution.

The RDFS schema defines the structural vocabulary of the ontology. Cardinality constraints, propagation rules, and compliance validation (e.g., mandatory quality tests, quarantine propagation) are implemented at the OWL rule and/or SHACL validation layer and are not encoded directly in the RDFS specification provided here.
